# Silencing of the Ca^2+^ Channel ORAI1 Improves the Multi-Systemic Phenotype of Tubular Aggregate Myopathy (TAM) and Stormorken Syndrome (STRMK) in Mice

**DOI:** 10.3390/ijms23136968

**Published:** 2022-06-23

**Authors:** Roberto Silva-Rojas, Laura Pérez-Guàrdia, Emma Lafabrie, David Moulaert, Jocelyn Laporte, Johann Böhm

**Affiliations:** 1 IGBMC (Institut de Génétique et de Biologie Moléculaire et Cellulaire), Inserm U1258, CNRS UMR7104, Université de Strasbourg, 67404 Illkirch, France; roberto.silva@cnic.es (R.S.-R.); perezgul@igbmc.fr (L.P.-G.); emma.lafabrie@etu.unistra.fr (E.L.); 2 Institut Clinique de la Souris (ICS), 67404 Illkirch, France; moulaert@igbmc.fr

**Keywords:** tubular aggregate myopathy, Stormorken syndrome, muscle disorder, calcium, STIM1, ORAI1, mouse model, ion channel, shRNA

## Abstract

Tubular aggregate myopathy (TAM) and Stormorken syndrome (STRMK) form a clinical continuum associating progressive muscle weakness with additional multi-systemic anomalies of the bones, skin, spleen, and platelets. TAM/STRMK arises from excessive extracellular Ca^2+^ entry due to gain-of-function mutations in the Ca^2+^ sensor STIM1 or the Ca^2+^ channel ORAI1. Currently, no treatment is available. Here we assessed the therapeutic potential of ORAI1 downregulation to anticipate and reverse disease development in a faithful mouse model carrying the most common TAM/STRMK mutation and recapitulating the main signs of the human disorder. To this aim, we crossed *Stim1^R304W/+^* mice with *Orai1^+/−^* mice expressing 50% of ORAI1. Systematic phenotyping of the offspring revealed that the *Stim1^R304W/+^Orai1^+/−^* mice were born with a normalized ratio and showed improved postnatal growth, bone architecture, and partly ameliorated muscle function and structure compared with their *Stim1^R304W/+^* littermates. We also produced AAV particles containing *Orai1*-specific shRNAs, and intramuscular injections of *Stim1^R304W/+^* mice improved the skeletal muscle contraction and relaxation properties, while muscle histology remained unchanged. Altogether, we provide the proof-of-concept that *Orai1* silencing partially prevents the development of the multi-systemic TAM/STRMK phenotype in mice, and we also established an approach to target *Orai1* expression in postnatal tissues.

## 1. Introduction

ORAI1, ORAI2, and ORAI3 are broadly expressed and highly selective calcium (Ca^2+^) channels residing at the plasma membrane. Owing to their primary role as regulators of extracellular Ca^2+^ influx, they were named after the three Horai, Eunomia, Dike, and Eirene, known as the guardians of the gates of Olympus in Greek mythology [1]. Ca^2+^ is a universal second messenger and initiates a wide variety of conserved signaling cascades. It is primarily stored in the endoplasmic/sarcoplasmic reticulum (ER/SR), and the transient increase of cytosolic Ca^2+^ levels modulates transcription and mediates a multitude of biological processes including cell proliferation and motility, exocytosis, nerve conduction, hormone release, coagulation, and muscle contraction [2]. Hence, the precise regulation of Ca^2+^ entry, Ca^2+^ storage, and Ca^2+^ release is fundamental for normal physiology in all cell types. One of the major mechanisms controlling Ca^2+^ homeostasis is store-operated Ca^2+^ entry (SOCE), which essentially relies on the concerted activity of the Ca^2+^ channel ORAI1 and the reticular Ca^2+^ sensor STIM1. Ca^2+^ store depletion from the ER/SR induces a conformational change of STIM1, resulting in protein oligomerization and the interaction with ORAI1 to trigger extracellular Ca^2+^ entry, ensure Ca^2+^ store refill, and maintain high Ca^2+^ gradients enabling oscillatory Ca^2+^ signaling [3,4].

Pathologic alterations of SOCE impeding or increasing Ca^2+^ influx profoundly compromise proper Ca^2+^ signaling and impact various molecular, physiological, and biochemical functions in tissues and organs, leading to multi-systemic mirror diseases [5,6]. Recessive *STIM1* and *ORAI1* loss-of-function (LoF) mutations inhibit SOCE and Ca^2+^ store refill and cause immunodeficiency (IMD9 and IMD10, OMIM # 612782, #612783), characterized by recurrent and chronic infections, autoimmunity, muscular hypotonia, mydriasis, and amelogenesis imperfecta [1,7,8]. By contrast, dominant *STIM1* and *ORAI1* gain-of-function (GoF) mutations induce SOCE overactivity and excessive Ca^2+^ entry and give rise to tubular aggregate myopathy (TAM) and Stormorken syndrome (STRMK) (OMIM #160565, #615883), two clinically overlapping disorders associating childhood-onset muscle weakness with miosis, ichthyosis, short stature, hyposplenism, thrombocytopenia, and dyslexia [9,10,11,12,13,14,15,16,17,18]. In analogy to the human disorders, mice either lacking *Stim1* or *Orai1*, or carrying GoF mutations in these genes respectively recapitulate the main clinical signs of immunodeficiency or TAM/STRMK [19,20,21,22], and represent valuable tools to investigate disease progression, uncover the underlying pathomechanisms, and identify therapeutic targets. Most *Orai1^−/−^* mice die perinatally, and the few surviving pups show defective B-cell and T-cell function and cytokine production, while heterozygous *Orai1^+/−^* animals are normal and fertile, demonstrating that the remaining *Orai1* expression of 50% is sufficient to ensure vital SOCE activity [22]. *Stim1^R304W/+^* mice harboring the most common TAM/STRMK mutation are smaller and weaker than their littermates, and manifest bone, platelet, spleen, and skin anomalies [21]. Histological analyses of *Stim1^R304W/+^* muscle sections revealed the presence of fibers with Ca^2+^ overload [21], and functional investigations in animals and on muscle extracts showed that the elevated cytosolic Ca^2+^ levels hamper regular muscle contraction and lead to sustained reticular stress, resulting in increased cell death and muscle fiber turnover [23]. Other TAM/STRMK mouse models harboring different *Stim1* missense mutations also exist, and they either show isolated anomalies of the platelets and muscles [19,24] or manifest an incomplete penetrance of the multi-systemic TAM/STRMK phenotype [25]. Tubular aggregates, although pathognomonic for the disease, are not found on muscle sections from TAM/STRMK mouse models [19,21,25].

There is currently no treatment for TAM/STRMK, but SOCE is susceptible to manipulation through modulation of ORAI1. To this aim, we crossed *Stim1^R304W/+^* with *Orai1^+/−^* animals, and the offspring underwent systematic phenotyping at the macroscopic and molecular levels. The *Stim1^R304W/+^Orai1^+/−^* mice showed improved body size and bone architecture, and partly ameliorated muscle function and structure compared with their *Stim1^R304W/+^* littermates. Based on this proof-of-concept illustrating the therapeutic potential of reduced *Orai1* expression, we implemented a practical method targeting *Orai1* after birth. Local injection of AAVs containing *Orai1*-specific shRNAs resulted in ameliorated muscle contraction and relaxation properties in TAM/STRMK mice but failed to improve muscle morphology. Overall, our data highlight ORAI1 downregulation as a suitable method to partially antagonize the multi-systemic TAM/STRMK phenotype.

## 2. Results

*Stim1^R304W/+^* mice recapitulate the main clinical signs of the human disorder [21], and the availability of a faithful animal model offers the possibility to establish and validate therapeutic approaches. To antagonize the development of TAM/STRMK, we crossed *Stim1^R304W/+^* mice [21] with *Orai1^+/−^* mice [22] expressing 50% of the Ca^2+^ channel ORAI1. The resulting WT, *Orai1^+/−^*, *Stim1^R304W/+^*, and *Stim1^R304W/+^Orai1^+/−^* offspring underwent comparative phenotyping to conclude on the therapeutic potential of *Orai1* downregulation. We assessed postnatal growth, overall development, bone architecture, spleen morphology, platelet numbers, and general muscle force at 1 to 4 months. In situ muscle force and skin cross sections were analyzed at 8 months since specific anomalies affecting both tissues occur at later disease stages in the *Stim1^R304W/+^* model [21].

### 2.1. Normalized Birth Ratio, and Improved Body Size and Weight Gain of Stim1^R304W/+^Orai1^+/−^ Mice

We previously reported that the number of *Stim1^R304W/+^* pups is below the expected Mendelian ratio and that the viable animals are smaller than their WT littermates throughout life [21], pointing to a crucial role of SOCE in prenatal and postnatal development. To assess whether *Orai1* downregulation prevents sporadic embryonic death and improves early growth stages, we crossed *Stim1^R304W/+^* with *Orai1^+/−^* mice and genotyped almost 300 offspring seven days after birth (Figure S1A). In line with WT (23%) and *Orai1^+/−^* (31%) animals, and compared with *Stim1^R304W/+^* mice (19%), *Stim1^R304W/+^Orai1^+/−^* pups were born with a normalized proportion of 27% (χ^2^ test, *p* = 0.036 n = 294). Extraction of skeletal muscle RNA and subsequent RT-qPCR evidenced a reduction of *Orai1* expression in *Orai1^+/−^* and *Stim1^R304W/+^Orai1^+/−^* mice compared with the WT and *Stim1^R304W/+^* littermates (Figure S1B), while *Orai2* and *Orai3* expression were comparable across all genotypes or slightly reduced (Figure S1C,D), confirming the deletion of an *Orai1* allele and ruling out a compensatory upregulation of its paralogues. In accordance with the RT-qPCR results, western blot on muscle extracts revealed reduced ORAI1 protein levels in *Stim1^R304W/+^Orai1^+/−^* mice compared with the WT and *Stim1^R304W/+^* littermates, while the STIM1 protein levels were comparable in all groups (Figure S1E–G).

**Figure 1:**
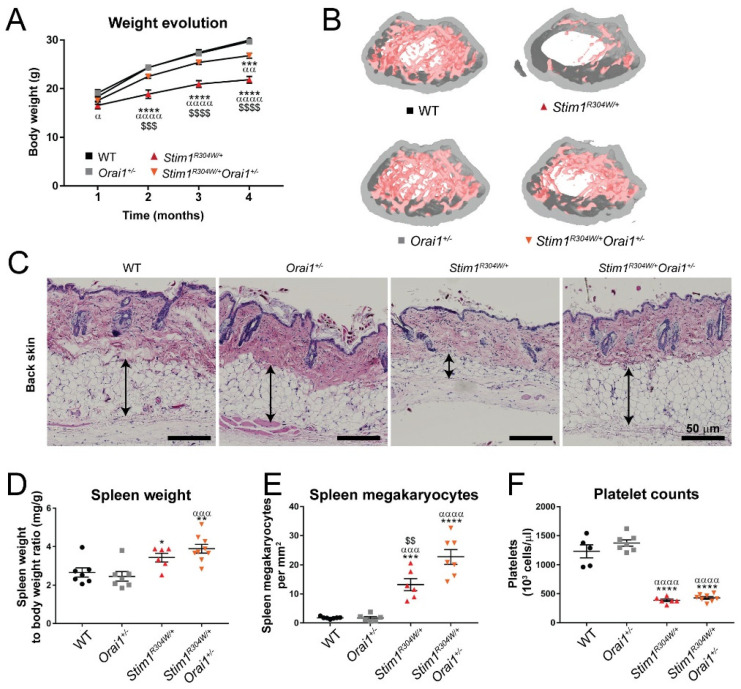
**Improved weight gain and bone structure in *Stim1^R304W/+^Orai1^+/−^* mice.** (**A**) Body weight evolution was ameliorated in *Stim1^R304W/+^Orai1^+/−^* mice compared with *Stim1^R304W/+^* littermates over the first months of life (n = 11–17). (**B**) Representative images of 3D reconstruction of the femur microarchitecture illustrated a similar trabecular density in *Stim1^R304W/+^Orai1^+/−^* mice and healthy WT and *Orai1^+/−^* controls. (**C**) Representative images showing histological H&E staining of back skin sections at 8 months. In total, 8 WT, 8 *Orai1^+/−^*, 6 *Stim1^R304W/+^*, and 6 *Stim1^R304W/+^Orai1^+/−^* mice were analyzed, and evidenced a normalized fat layer thickness (arrows) in four out of six *Stim1^R304W/+^Orai1^+/−^* mice (see Figure S2F). (**D**–**F**) Relative spleen weight, megakaryocyte numbers, and platelet counts were comparable in *Stim1^R304W/+^* and *Stim1^R304W/+^Orai1^+/−^* mice and significantly differed from the healthy controls (n = 5–9). Graphs represent mean ± SEM. Significant differences are indicated as */α/$ *p* < 0.05, **/αα/$$ *p* < 0.01, ***/ααα/$$$ *p* < 0.001, and ****/αααα/$$$$ *p* < 0.0001 with * reflecting the comparison with the WT group, α the comparison with the *Orai1^+/−^* group, and $ the comparison with the *Stim1^R304W/+^Orai1^+/−^* group.

We followed the body size and weight development of the offspring over 4 months and in accordance with our previous studies [21], the *Stim1^R304W/+^* mice showed a flatter growth curve in comparison with the control littermates (Figure 1A). At every time point of measurement, the *Stim1^R304W/+^Orai1^+/−^* mice were significantly bigger and heavier than the *Stim1^R304W/+^* mice with a difference of 75 mm and 5 g at 4 months, corresponding to an increase of 23% and 10%, respectively (Figure 1A and Figure S2A). Overall, our data confirmed the lower birth ratio and weight gain of *Stim1^R304W/+^* mice and the absence of an overt deleterious effect of ORAI1 downregulation in *Orai1^+/−^* mice. The data also suggest that *Stim1^R304W/+^Orai1^+/−^* offspring overcome the risk of perinatal lethality and document an ameliorated postnatal development of the TAM/STRMK animals with reduced *Orai1* expression.

### 2.2. Improved Bone Architecture in Stim1^R304W/+^Orai1^+/−^ Mice

The continuous growth of organisms from birth to adulthood is intrinsically linked to the counterbalance of bone-forming osteoblasts and bone-resorbing osteoclasts, and the proliferation and differentiation of both osteoblasts and osteoclasts are SOCE-dependent [26,27]. Consistently, *Stim1^R304W/+^* bones were shown to exhibit structural anomalies of the bones [21], presumably accounting for the short stature of TAM/STRMK patients and mice. To determine if the ameliorated growth curves of *Stim1^R304W/+^Orai1^+/−^* mice correlate with proper bone architecture, we performed micro-computerized tomography to obtain 3D representations. Bones from *Stim1^R304W/+^Orai1^+/−^* animals showed an improved cortical and trabecular texture and strength compared with *Stim1^R304W/+^* mice as illustrated by a significantly increased moment of inertia (MOI) of 33% and a reduced trabecular separation of 43% of tibia and femur, respectively (Figure 1B and Tables S3 and S4).

### 2.3. Unchanged Skin, Spleen, and Platelet Phenotypes in Stim1^R304W/+^Orai1^+/−^ Mice

Skin anomalies including ichthyosis, eczema, or anhidrosis are common features of TAM/STRMK [13]. Histological analyses of patient samples disclosed an obstruction of the eccrine glands, resulting in sweat retention and representing a risk factor for associated skin irritations [28], and *Stim1^R304W/+^* mice displayed an enlarged dermis and a thinning of the subcutaneous fat layer [21]. To evaluate the impact of *Orai1* downregulation on dermal composition, we examined cross sections of *Stim1^R304W/+^* and *Stim1^R304W/+^Orai1^+/−^* skin samples at 8 months. Although four out of six *Stim1^R304W/+^Orai1^+/−^* mice showed a distinct increase in the fat layer area, no overall significant difference was measurable compared to *Stim1^R304W/+^* mice (Figure 1C and Figure S2B).

Another hallmark of TAM/STRMK is spleen dysfunction in combination with thrombocytopenia and bleeding diathesis [6,8,10,12,29]. Alike the human phenotype, *Stim1^R304W/+^* mice showed morphological spleen anomalies and a reduction of the total platelet number by 70% [21], resulting in reduced thrombus formation upon injury and increased bleeding times. *Stim1^R304W/+^Orai1^+/−^* animals also manifested splenomegaly and prominent hyperplasia of the megakaryocytes, the precursor cells forming and releasing platelets into the bloodstream (Figure 1D,E). In compliance with the uncorrected spleen phenotype, platelet counts were similarly low in *Stim1^R304W/+^* and *Stim1^R304W/+^Orai1^+/−^* animals and associated with increased bleeding times (Figure 1F and Figure S2C), indicating that the downregulation of *Orai1* by 50% has no reversing effect on the spleen and platelet anomalies characterizing TAM/STRMK.

### 2.4. Improved Muscle Contraction Properties in Stim1^R304W/+^Orai1^+/−^ Mice

**Figure 2:**
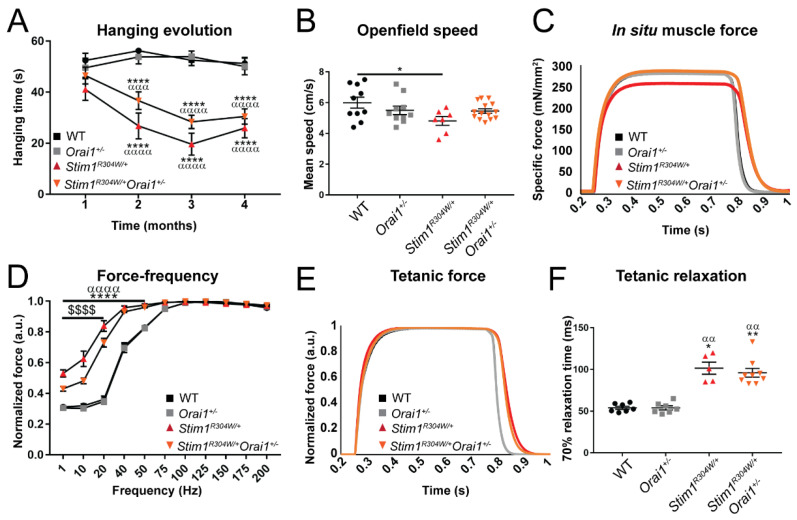
**Partially improved muscle performance of *Stim1^R304W/+^Orai1^+/−^* mice.** (**A**) *Stim1^R304W/+^Orai1^+/−^* mice showed a continuous but not significant tendency of increased hanging times compared with *Stim1^R304W/+^* littermates between 1 and 4 months (n = 11–17). (**B**) The velocity of *Stim1^R304W/+^Orai1^+/−^* mice in the open field arena was indistinguishable from WT and *Orai1^+/−^* controls at 10 weeks of age and slightly but not significantly improved compared with *Stim1^R304W/+^* littermates (n = 7–14). (**C**) In situ measurements at 8 months revealed an increased specific muscle force of *Stim1^R304W/+^Orai1^+/−^* compared with *Stim1^R304W/+^* mice at 100 Hz (n = 5–7). (**D**–**F**) Stimulation frequencies of 1–200 Hz evidenced a significant shift of the *Stim1^R304W/+^Orai1^+/−^* muscle contraction properties towards normal values, while muscle relaxation following tetanic stimulation at 100 Hz was similar in *Stim1^R304W/+^* and *Stim1^R304W/+^Orai1^+/−^* mice (n = 5–9) The curves in 2E are normalized to facilitate the comparison of muscle contraction and relaxation kinetics. Graphs represent mean ± SEM. Significant differences are indicated as */α/$ *p* < 0.05, **/αα/$$ *p* < 0.01, ***/ααα/$$$ *p* < 0.001, and ****/αααα/$$$$ *p* < 0.0001 with * reflecting the comparison with the WT group, α the comparison with the *Orai1^+/−^* group, and $ the comparison with the *Stim1^R304W/+^Orai1^+/−^* group.

Muscle weakness and exercise intolerance are major disabling traits of TAM/STRMK [13]. Affected individuals have difficulties climbing stairs, running, or standing up from a squatting position, and consistently, *Stim1^R304W/+^* mice manifest deficiencies in general and specific muscle force [21]. The initial characterization of the animal model did not reveal any differences of in situ muscle force upon nerve and muscle stimulation, ruling out a transmission defect at the neuromuscular junction (NMJ) as the cause of the muscle weakness [21]. To assess a potential improvement of muscle performance through *Orai1* downregulation, *Stim1^R304W/+^Orai1^+/−^* and control mice underwent hanging and open field tests at 4 months complemented by force transduction experiments at 8 months. Compared with their *Stim1^R304W/+^* littermates, *Stim1^R304W/+^Orai1^+/−^* mice showed non-significant tendencies of increased hanging times throughout the first 4 months (Figure 2A) and higher mean speed and covered distance in the open field at 3 months (Figure 2B and Figure S2D). In situ muscle force measurements on tibialis anterior at 8 months of age revealed an increased specific muscle force and a tendency of a higher maximal force of *Stim1^R304W/+^Orai1^+/−^* compared with *Stim1^R304W/+^* mice (Figure 2C and Figure S2E).

Muscle contraction is a multistep process initiated by an electrical stimulus and mediated by the release of Ca^2+^ from the SR. The Ca^2+^ ions trigger the shortening of the contractile units to generate force [30], and Ca^2+^ store refill through the ATP-dependent SERCA pumps enables muscle relaxation and maintains high Ca^2+^ gradients across the SR membrane to allow repetitive tetanic stimulations and counteract the effects of fatigue [31,32]. As a consequence of the Ca^2+^ abundance at the contractile units, *Stim1^R304W/+^* mice manifest an increased force production at low stimulation frequencies together with a delay in muscle contraction and muscle relaxation, which results in abnormal fatigue profiles and possibly corresponds to the muscle cramping phenotype observed in TAM/STRMK patients [10,21,23]. In *Stim1^R304W/+^Orai1^+/−^* mice, the force production between 1 and 20 Hz and the muscle contraction kinetics following a single impulse distinctively shifted towards the WT values without reaching normalization (Figure 2D–F and Figure S3A,B), and we also noted a non-significant tendency of ameliorated muscle relaxation (Figure S3A,C). The fatigue curves following repetitive stimulations however remained identical in *Stim1^R304W/+^Orai1^+/−^* and *Stim1^R304W/+^* mice, suggesting unresolved muscle cramping (Figure S3D–G). In summary, the reduction of *Orai1* expression by half has measurable and in parts significant effects on specific muscle force and functionality parameters in *Stim1^R304W/+^Orai1^+/−^* mice.

### 2.5. Normalized Muscle Fiber Size and Improved Autophagy Markers in Stim1^R304W/+^Orai1^+/−^ Mice

**Figure 3:**
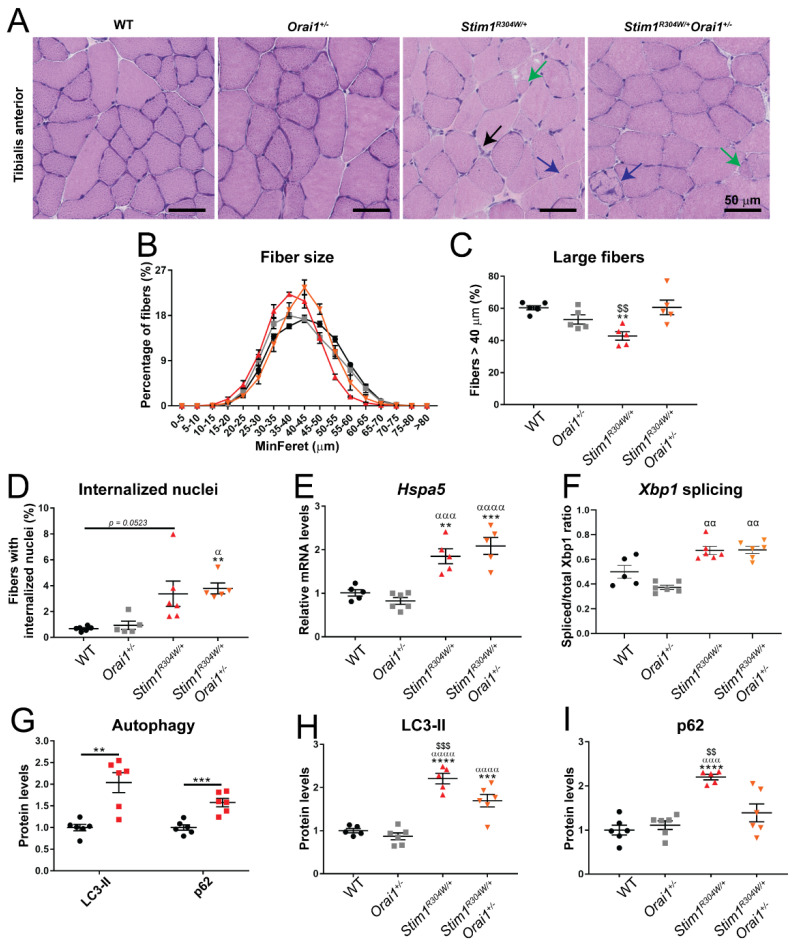
**Increased myofiber size and improved autophagy markers in *Stim1^R304W/+^Orai1^+/−^* mice.** (**A**) Representative images of H&E staining on muscle sections from both *Stim1^R304W/+^* and *Stim1^R304W/+^Orai1^+/−^* mice at 4 months revealed signs of muscle fiber degeneration such as centralized nuclei (blue arrows), regenerating fibers (green arrow), and immune cell infiltrations (black arrows). (**B**,**C**) Different fiber size distribution in *Stim1^R304W/+^* and *Stim1^R304W/+^Orai1^+/−^* mice and normalization of fibers with a MinFeret diameter of >40 µm in *Stim1^R304W/+^Orai1^+/−^* muscle at 4 months (n = 5 mice per group). (**D**–**F**) Quantification revealed a comparable number of fibers with centralized nuclei and similar expression levels/splicing ratios of the UPR markers *Hspa5* and *XbpI* in *Stim1^R304W/+^* and *Stim1^R304W/+^Orai1^+/−^* muscle at 4 months (n = 5–6). (**G**) Increased LC3-II and p62 protein levels in *Stim1^R304W/+^* muscle samples compared to WT at 4 months (n = 6 mice per group). (**H**,**I**) Reduced LC3-II and normalized p62 protein levels in *Stim1^R304W/+^Orai1^+/−^* muscle samples compared to *Stim1^R304W/+^* mice at 4 months (n = 5–6). Graphs represent mean ± SEM. Significant differences are indicated as */α/$ *p* < 0.05, **/αα/$$ *p* < 0.01, ***/ααα/$$$ *p* < 0.001, and ****/αααα/$$$$ *p* < 0.0001 with * reflecting the comparison with the WT group, α the comparison with the *Orai1^+/−^* group, and $ the comparison with the *Stim1^R304W/+^Orai1^+/−^* group.

Muscle weakness in TAM/STRMK mice is accompanied by myofiber atrophy and signs of muscle fiber degeneration and regeneration on biopsies such as nuclear centralization and infiltration of immune cells [21,23]. To determine if the improved muscle performance of *Stim1^R304W/+^Orai1^+/−^* mice bears on an ameliorated muscle structure, we histologically analyzed transverse tibialis anterior sections. *Stim1^R304W/+^Orai1^+/−^* muscle samples displayed an overall enlargement of fiber caliber with 61% of the fibers exceeding a MinFeret diameter of 40 µm—corresponding to the median myofiber diameter in 4-month-old WT mice—compared with 43% in *Stim1^R304W/+^* littermates (Figure 3A–C). However, the number of fibers with central nuclei was still raised in *Stim1^R304W/+^Orai1^+/−^* tibialis anterior, indicating persistent myofiber degeneration (Figure 3D).

To assess whether muscle fiber degeneration was concomitant with continuous regeneration, we determined the overall satellite cell number and activation status through immunofluorescence. The ratio of activated KI-67-positive satellite cells was increased in muscle samples from *Stim1^R304W/+^* mice compared with the control littermates (Figure S4A,B), confirming a sustained regeneration process. Of note, satellite cell activation was moderately but significantly lower in *Stim1^R304W/+^Orai1^+/−^* mice compared with *Stim1^R304W/+^* animals, highlighting a measurable effect of ORAI1 downregulation on muscle integrity.

To explore the mechanisms underlying the increase of myofiber diameter in *Stim1^R304W/+^Orai1^+/−^* mice, we addressed autophagy, an organelle recycling pathway implicated in the regulation of muscle mass [33]. We detected a comparable or slightly decreased expression of the main autophagy genes *Map1lc3a*, *Map1lc3b*, and *Sqstm1* in *Stim1^R304W/+^* mice compared with the WT (Figure S5A), while western blots on muscle extracts revealed an increased level of the autophagosome components LC3-II and p62 (Figure 3G and Figure S5B,C), indicating enhanced autophagosome formation or impaired fusion with the lysosome and suggesting a block of late-stage autophagy. Noteworthy, the LC3-II and p62 levels were significantly reduced in *Stim1^R304W/+^Orai1^+/−^* tibialis anterior compared with *Stim1^R304W/+^* mice (Figure 3H,I and Figure S5E), indicating a partial recovery of the autophagic flux through *Orai1* downregulation and providing a potential explanation for the increase in muscle fiber diameter despite continued myofiber degeneration.

Muscle fiber degeneration in *Stim1^R304W/+^* mice results from Ca^2+^-induced reticular stress and the activation of unfolded protein response (UPR) and apoptosis pathways [23]. RT-qPCR on selected UPR markers revealed a comparable upregulation of the chaperones *Hspa5/Bip1* and *Hsp90b1* in the tibialis anterior of both *Stim1^R304W/+^* and *Stim1^R304W/+^Orai1^+/−^* animals (Figure 3E and Figure S6), suggesting that reticular stress as the origin of myofiber degeneration was not resolved in *Stim1^R304W/+^Orai1^+/−^* muscle. This was confirmed by an increased splicing ratio of the *Xbp1* transcription factor in *Stim1^R304W/+^* and *Stim1^R304W/+^Orai1^+/−^* muscle samples (Figure 3F), leading to the translation of the XBP1s isoform, implicated in the transcriptional regulating of UPR target genes [34].

### 2.6. shRNA-Driven Orai1 Silencing Partially Reverses the Muscle Phenotype of Stim1^R304W/+^ Mice

The crossing experiments on our TAM/STRMK mouse model and the survey of birth ratio, growth, and bone, skin, spleen, platelet, and muscle phenotypes of the *Stim1^R304W/+^Orai1^+/−^* offspring and control littermates provided the proof-of-concept that decreased *Orai1* expression partially anticipates full disease development with a discernible impact on skeletal muscle function and structure. To establish an appropriate and applicable procedure to specifically downregulate *Orai1* in postnatal tissues, we used RNA interference.

We aligned the mouse *Orai1* sequence with its paralogues *Orai2* and *Orai3*, and we designed four shRNAs targeting stretches of 19 to 22 *Orai1*-specific nucleotides largely conserved in humans (Figure S7). Transfection of murine C2C12 myoblasts and subsequent RNA extraction and RT-qPCR demonstrated an *Orai1* downregulation of at least 50% through shRNAs sh22, sh190, and sh760 compared with untransfected controls or cells expressing scramble shRNAs (Figure S7). To validate *Orai1* silencing in vivo, we generated AAV9 particles containing the shRNAs and injected the tibialis anterior of 1-month-old WT mice. Four weeks post-injection, sh22 and sh190 yielded an *Orai1* downregulation of more than 80% as compared to NaCl-injected control muscles, while sh760 was less efficient and therefore discarded (Figure S7).

**Figure 4:**
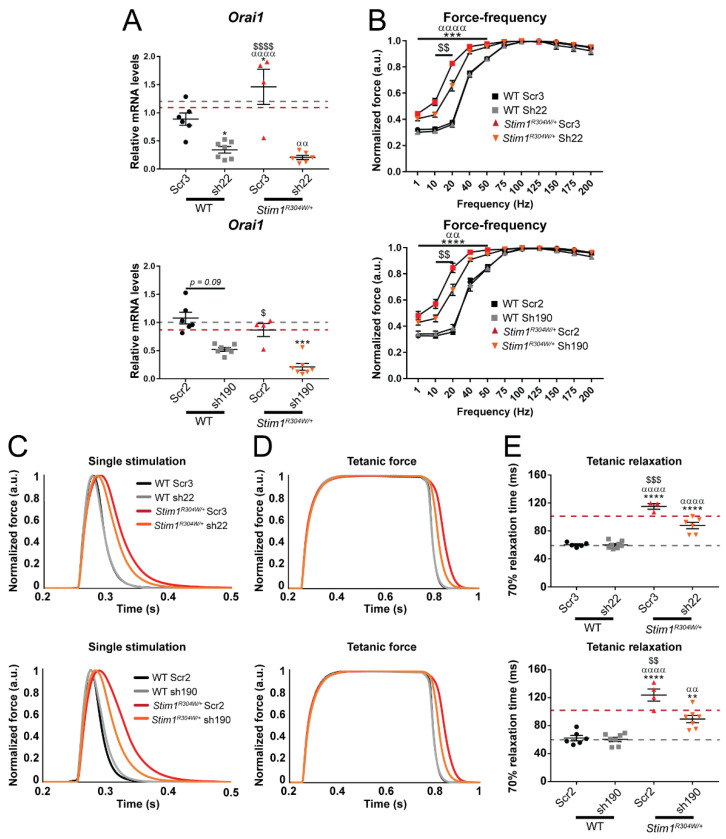
**Improved muscle contraction and relaxation properties in TAM/STRMK mice through *Orai1* silencing 2 months post shRNA injection**. (**A**) sh22 (top) and sh190 (bottom) yielded a 80% decrease of *Orai1* expression in *Stim1^R304W/+^* muscle compared with scramble-injected WT, NaCl-injected WT (black dashed line), and NaCl-injected *Stim1^R304W/+^* (red dashed line) controls (n = 4–7). (**B**) Shifted force production towards normal values at low stimulation frequencies in *Stim1^R304W/+^* tibialis anterior treated with sh22 (top) and sh190 (bottom) compared with scramble-injected controls (n = 4–8). NaCl-injected controls are not shown. (**C**,**D**) Improved muscle relaxation after single and tetanic (100 Hz) stimulation of *Stim1^R304W/+^* tibialis anterior injected with sh22 (top) and sh190 (bottom) compared with scramble-injected controls (n = 3–8). (**E**) The time required for a muscle relaxation of 70% is significantly reduced in *Stim1^R304W/+^* tibialis anterior injected with sh22 (top) and sh190 (bottom) compared with scramble-injected controls, NaCl-injected WT (black dashed line), and NaCl-injected *Stim1^R304W/+^* mice (red dashed line) (n = 3–8). Graphs represent mean ± SEM. Significant differences are indicated as */α/$ *p* < 0.05, **/αα/$$ *p* < 0.01, ***/ααα/$$$ *p* < 0.001, and ****/αααα/$$$$ *p* < 0.0001 with * reflecting the comparison with the scramble-injected WT group, α the comparison with the shRNA-injected WT group, and $ the comparison with the scramble-injected *Stim1^R304W/+^* group.

To determine the ability of the selected shRNAs to reverse the muscle defects of TAM/STRMK, we proceeded with the intramuscular AAV injection of either sh22 or sh190 in WT and *Stim1^R304W/+^* mice at 2 months of age, and we investigated muscle function, structure, and physiology 2 months post injection. *Orai1* downregulation ranged from 50% to 80% (Figure 4A), whereas the expression levels of *Orai2* and *Orai3* were comparable in the shRNA-injected, NaCl-injected, and scramble-injected muscles (Figure S8A–D), demonstrating high specificity of the shRNAs. In situ measurements on anesthetized animals showed a positive effect of both sh22 and sh190 on the force production at low stimulation frequencies (especially 10 and 20 Hz) of *Stim1^R304W/+^* mice compared with the scramble shRNAs, while the muscle contraction properties did not vary between shRNA-injected and NaCl-injected WT mice, excluding a negative impact of the shRNAs on normal muscle function (Figure 4B). We also observed an improvement of the muscle relaxation kinetics with reduced relaxation times in *Stim1^R304W/+^* tibialis anterior injected with sh22 and sh190 following single and tetanic stimulations (Figure 4C–E).

Histological examination of *Stim1^R304W/+^* tibialis anterior sections failed to disclose ameliorations of the muscle structure following shRNA delivery. The proportion of fibers with a MinFeret diameter of >55 µM and the number of fibers with centralized nuclei were comparable in shRNA and scramble-injected *Stim1^R304W/+^* muscles (Figure S9A–D). In agreement with the morphological findings, there was no difference in the expression levels of UPR and autophagy markers in *Stim1^R304W/+^* tibialis anterior treated with sh22, sh190, or scramble shRNAs (Figures S10A–D and S11). Overall, the acute shRNA-mediated downregulation of *Orai1* over a postnatal period of 8 weeks did not resolve reticular Ca^2+^ stress and autophagy block, but partially improved muscle contraction and relaxation properties of the murine TAM/STRMK model.

## 3. Discussion

**Figure 5:**
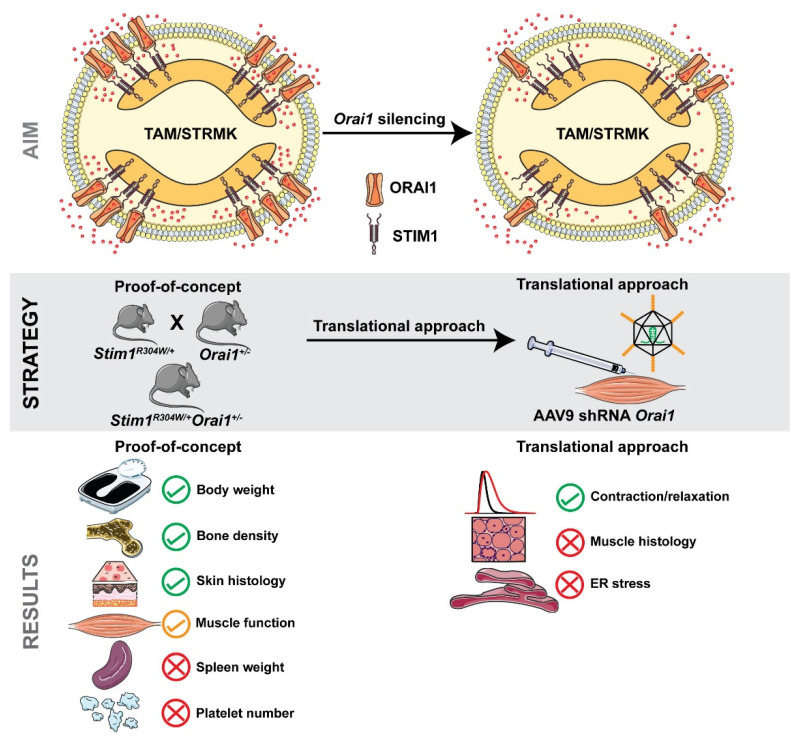
**Graphical overview.** Illustration of the aim, experimental strategy, and main results of the present study.

Tubular aggregate myopathy (TAM) and Stormorken syndrome (STRMK) are spectra of the same multi-systemic disease affecting muscle, bones, skin, muscles, spleen, and skin [10]. They are caused by gain-of-function mutations in *STIM1* and *ORAI1*, encoding key components of the ubiquitous store-operated Ca^2+^ entry (SOCE) mechanism [13]. There is currently no treatment for TAM/STRMK, and here we provide the proof-of-concept that the genetic downregulation of the Ca^2+^ entry channel ORAI1 partially improves the multi-systemic phenotype in a faithful mouse model of the disorder. In addition, we specifically targeted *Orai1* expression through AAV-mediated delivery of shRNAs in murine TAM/STRMK muscle and thus furnished a method to downregulate ORAI1 after birth. A graphical overview of the experimental design and the main results is provided in Figure 5.

### 3.1. ORAI1 as the Main Target to Treat the Multi-Systemic TAM/STRMK Phenotype

Store-operated Ca^2+^ entry (SOCE) is an essential mechanism controlling Ca^2+^ influx in all tissues and organs to regulate countless Ca^2+^-dependent metabolic processes, signaling pathways, and cellular functions. By way of example, SOCE drives osteoblastogenesis and osteoclastogenesis and thereby governs the dynamic balance of bone deposition and bone resorption required for growth [26,35,36]. SOCE also activates blood clotting following injury through Ca^2+^-dependent secretion of alpha granules from platelets to induce thrombus formation [37,38], directs the differentiation and migration of keratinocytes in the epidermis [39,40], and triggers the opening of a Ca^2+^-activated chloride channel for sweat production [41]. Furthermore, efficient muscle contraction is predicated on the precise control of Ca^2+^ flows between the SR and the cytosol, and the SOCE-mediated Ca^2+^ store refill counteracts the effects of fatigue [31,32]. As a consequence, the dysfunction of SOCE and its principal elements STIM1 and ORAI1 severely interferes with Ca^2+^ homeostasis and compromises normal physiology in multiple tissues [42].

Considering that TAM/STRMK arises from excessive extracellular Ca^2+^ influx, the downregulation of the Ca^2+^ entry channel ORAI1 appears as a straightforward approach to attenuate or reverse the multi-systemic anomalies of bones, skin, spleen, platelets, and muscle. Moreover, ORAI1 acts downstream of the other known TAM/STRMK genes and hence represents the most appropriate target for a common therapy of all disease forms. Indeed, the overall reduction of available ORAI1 monomers to shape functional channels hexamers will at least partially mitigate the effects of *ORAI1* mutations generating a leaky channel [43], of *STIM1* mutations inducing constitutive ORAI1 opening [9,12,14,15], and of *CASQ1* mutations interfering with STIM1 retention and the negative regulation of SOCE [44,45]. This is supported by a previous study showing that the dystrophic phenotype of transgenic mice overexpressing WT STIM1 is improved by a dominant-negative ORAI1 mutant [46].

### 3.2. Orai1 Downregulation Improves Several but Not All Multi-Systemic TAM/STRMK Phenotype

The *Stim1^R304W/+^* mouse replicates the multi-systemic phenotype of the human disorder [21] and represents an adequate model to assess preclinical therapies. Here we crossed our *Stim1^R304W/+^* model with *Orai1^+/−^* mice to obtain *Stim1^R304W/+^Orai1^+/−^* offspring carrying the most common TAM/STRMK mutation and expressing only 50% of the Ca^2+^ entry channel ORAI1. Of note, the total knockout of *Orai1* in mice is lethal [22], and the tissue-specific deletion of *Orai1* or the generation of chimeras through transplantation of hematopoietic *Orai1^−/−^* stem cells results in defective T cell activation in response to antigens [22,47], reduced platelet activation and thrombus formation [48], anhidrosis [41], amelogenesis imperfecta [22], and muscle weakness [49]. Accordingly, patients carrying homozygous *ORAI1* LoF mutations abolishing SOCE manifest immunodeficiency associated with skin anomalies, ectodermal dysplasia, and muscular hypotonia [1], emphasizing the importance of operative SOCE for normal development. However, heterozygous carriers of immunodeficiency mutations are healthy, and mice deprived of a single *Orai1* allele do not show any apparent pathology, demonstrating that the remaining *Orai1* expression of 50% is sufficient to preserve the necessary SOCE activity in immune, skin, blood, ectoderm, and muscle cells.

Phenotyping of the *Stim1^R304W/+^Orai1^+/−^* mice from birth to the age of 4 months revealed a rescue of the birth ratio, a significant improvement of growth and weight development, and bone architecture, and a partial amelioration of muscle function and structure compared with *Stim1^R304W/+^* mice fully expressing *Orai1*. However, the skin and spleen phenotypes were not relieved, and *Stim1^R304W/+^Orai1^+/−^* mice displayed the same thrombocytopenia and coagulation defects as their TAM/STRMK littermates. This is possibly due to the disparate regulation of SOCE fine-tuning in the different cell types forming an organism. Fibroblasts, lymphocytes, macrophages, megakaryocytes, or platelets dispose of specific sets of SOCE modulators [50] and might be less responsive to changes in *Orai1* expression than osteoblasts or muscle fibers. Alternatively, the ORAI1 paralogues ORAI2 and ORAI3 or other Ca^2+^ channels as the TRPCs may adopt a leading role in the regulation of SOCE in skin, spleen, and platelets, and thereby dilute the effect of *Orai1* downregulation.

### 3.3. shRNA-Mediated Silencing of Orai1 Partially Improved Muscle Function

The rescue of birth ratio and the improvement of postnatal development of *Stim1^R304W/+^Orai1^+/−^* offspring as exemplified by growth, bone structure, and muscle contractibility illustrate the therapeutic potential of *Orai1* reduction for TAM/STRMK. Based on this proof-of-concept, we aimed to establish a practical method to downregulate *Orai1* in our murine *Stim1^R304W/+^* model and assess the reversal of the TAM/STRMK phenotype in postnatal muscle. We designed *Orai1*-specific shRNAs with homology to the human *ORAI1* sequence, validated their effectiveness in cells, and delivered the most capable shRNAs via local AAV injections into the tibialis anterior of WT and *Stim1^R304W/+^* mice. We noticed improved muscle contraction and relaxation kinetics in transduced *Stim1^R304W/+^* animals, but no effect of the shRNAs on muscle morphology was observed—despite the reduction of *Orai1* expression by more than 50%. This is possibly due to the time point of treatment at 2 months and after the onset of the disease signs, resulting in the inability to revert structural anomalies of the myofibers once established, or may be related to the physiology of skeletal muscle. Indeed, myofibers have a comparatively low turnover by contrast with monocytes or intestinal epithelial cells for instance [51,52]. The full therapeutic effect of the selected shRNA may therefore be visible several weeks following AAV injection and possibly beyond the incubation period of 2 months. However, Ca^2+^ stress-induced UPR and structural muscle anomalies such as internalized nuclei were neither rescued in shRNA-treated *Stim1^R304W/+^* tibialis anterior nor in *Stim1^R304W/+^Orai1^+/−^* mice, suggesting that other limiting factors than fiber turnover account for the absence of rescuing effects on reticular stress and myofiber degeneration.

At least the blockage of autophagic flux appeared to be partially resolved in *Stim1^R304W/+^Orai1^+/−^* mice and provides a potential explanation for the increased number of larger fibers and the gain of muscle mass compared with *Stim1^R304W/+^* mice. It also suggests that the treatment with activators of autophagy such as trans-resveratrol, spermidine, or AICAR and mTORC1 inhibitors (RAD001/AZD8055) may be beneficial for TAM/STRMK patients to increase muscle force. Indeed, the administration of these compounds has previously been shown to restore the autophagy defects in murine models of Duchenne muscular dystrophy (DMD), collagen VI-related muscular dystrophies, and X-linked centronuclear myopathy (XLCNM) [53,54,55]. In a similar way, treatment with the chemical chaperone 4-PBA may overcome UPR and thereby anticipate the effects of Ca^2+^ stress and raise myofiber survival. This approach was proved to be effective in mouse models for DMD [56] and central core disease (CCD) [57], another muscle disorder involving cytosolic Ca^2+^ overload and reticular stress [58,59,60]. Furthermore, the SOCE inhibitors CIC-37 and CIC39 were recently shown to attenuate extracellular Ca^2+^ entry in cellular TAM/STRMK models [61], and other molecules acting on SOCE currently are undergoing clinical trials on medical conditions including asthma, cancer, pancreatitis, and psoriasis [62]. Taken together, the pharmacological treatment with general autophagy activators, chaperones, or chemical Ca^2+^ channel blockers may complement the more specific shRNA-mediated *Orai1* downregulation for a synergistic therapeutic outcome.

### 3.4. Concluding Remarks

Overall, our data on *Stim1^R304W/+^Orai1^+/−^* mice evidenced a physiological benefit of constitutive *Orai1* downregulation on a subset of the multi-systemic phenotypes characterizing TAM/STRMK with a measurable effect on body size and weight development, bone architecture, and a partial improvement of muscle function. We also established a practical approach using AAV-mediated delivery of shRNAs specifically and efficiently reducing *Orai1* expression in postnatal tissues, and we observed ameliorated but not normalized muscle contraction properties in *Stim1^R304W/+^* mice after a treatment period of 8 weeks. As a perspective, it remains to be determined if the systemic delivery of the *Orai1*-specific shRNAs in mice efficiently antagonizes and reverts the multi-systemic signs of TAM/STRMK, and whether this approach may serve therapeutic purposes in patients with TAM/STRMK and other Ca^2+^-related diseases.

## 4. Materials and Methods

### 4.1. Animals

Mice were housed in ventilated cages with 12 h day/night cycles and access to food and water ad libitum. *Stim1^R304W/+^* and *Orai1^+/−^* mice were described previously [21,22], and the *Orai1^+/−^* mice were a kind gift from Paul F. Worley (Johns Hopkins University, Baltimore, MD, USA). Crossing of both mouse lines resulted in four genotypes: WT, *Orai1^+/−^*, *Stim1^R304W/+^*, and *Stim1^R304W/+^Orai1^+/−^*. Owing to the more pronounced muscle weakness in males compared with female *Stim1^R304W/+^* mice, only males were used in the study. All animal experimentation was performed after weaning. The following primers were used for genotyping: GCAGGTAGGAGAGTGTACAGGATGCCTT (forward) and CTTTCCATCCCCACTGCCATTTT (reverse) for *Stim1*, and ATGCCTACTGCCAAAATTGAC (forward) and AAATACTGAGCCATCTCTCCTG (reverse) for *Orai1*.

### 4.2. Hanging and Open Field Tests

To assess general muscle force, mice were suspended upside down to a cage grid for a maximum of 60 s, and the hanging time was recorded. Hanging tests were performed monthly and in triplicate with a 5–10 min rest interval.

The open field test was performed on 3-month-old mice in a homogenously illuminated arena (Bioseb, Vitrolles, France) in a noise-isolated room. Covered distance, speed, and rearing were assessed for 30 min.

### 4.3. In Situ Muscle Force

To determine maximal and specific muscle force, 4- and 8-month-old mice were anesthetized with intraperitoneal injections of domitor/fentanyl mix (2/0.28 mg/Kg), diazepam (8 mg/Kg), and fentanyl (0.28 mg/Kg). The tibialis anterior (TA) was partially excised and the tendon was attached to the isometric transducer of the in situ whole animal system 1305A (Aurora Scientific, Aurora, ON Canada). The maximal force was determined by sciatic nerve stimulations of 2–200 Hz pulses with an interval of 30 s, and fatigue by 80 stimulations of 40 Hz spaced by 2 s. Specific muscle force was assessed by dividing the maximal force with the TA cross-sectional area calculated as wet muscle weight (mg)/optimal muscle length (mm) X mammalian muscle density (1.06 mg/mm^3^).

### 4.4. Micro-Computerized Bone Tomography (µCT)

Trabecular and cortical bone morphology and structure were assessed on femur and tibia using the Quantum µCT scanner (Perkin Elmer, Waltham, MA, USA). Scans were performed with 10 µm voxel size, 160 µA tube current, and 90 kV tube voltage. Grayscale images were pre-processed using the ImageJ software, and morphological 3D measurements were executed with the CTAn software (Bruker, Billerica, MA, USA). Representative images were generated using the CTvol software (Bruker).

### 4.5. Bleeding Test and Blood Counts

Mice were anesthetized by inhalation of isoflurane through masks. A distal 10 mm segment of the tail was amputated with a scalpel, and the tail was immediately immersed in 0.9% isotonic PBS solution at 37 °C. The bleeding time was defined as the time required until bleeding ceased. The blood-PBS solution underwent OD analysis to determine overall blood loss.

Blood counts were performed on the ADVIA 120 system (Siemens, Munich, Germany) following submandibular puncture under isoflurane anesthesia of 4–month-old mice to determine total platelet, erythrocyte, and leukocyte numbers.

### 4.6. Muscle, Spleen, and Skin Histology

TA muscles were frozen in liquid nitrogen-cooled isopentane and transverse 8 µm sections were stained with hematoxylin and eosin (H&E), and the Cellpose algorithm [63] was used to segment and delineate the individual myofibers. The MinFeret diameter was calculated using ImageJ (https://imagej.nih.gov/ij/, accessed on 23 April 2022), and the number of fibers with internal nuclei was determined through the Cell Counter ImageJ plugin. The spleen and a dorsal skin fragment were fixed in 4% paraformaldehyde for 24 h, embedded in paraffin, and 5 µm sections were stained with H&E. The megakaryocyte number was determined on random images covering 12.3 mm^2^ per spleen using the ImageJ Cell Counter plugin, and the thickness and relative proportion of the subcutaneous fat layer was determined on a 5 mm^2^ skin sample area using the NDP Viewer software (Hamamatsu, Hamamatsu, Japan). All muscle, spleen, and skin sections were imaged with the Nanozoomer 2HT slide scanner (Hamamatsu).

### 4.7. Gene Expression and Protein Studies

Total RNA was extracted from TA samples with TRI Reagent (Molecular Research Center, Cincinnati, OH, USA) and reverse transcribed using the SuperScriptTM IV Transcriptase (ThermoFisher Scientific, Waltham, MA, USA). For quantitative PCR, the cDNA was amplified using the SYBR Green Master Mix I (Roche Diagnostics, Basel, Switzerland) on a LightCycler 480 Real-Time PCR System (Roche) with forward and reverse primers (Table S1). Primer specificity was determined through melting curve products followed by Sanger sequencing of the amplicons. Rpl27 was used as reference gene [64].

For protein studies, TA cryosections were lysed in RIPA (radioimmunoprecipitation) buffer supplemented with 1 mM PMSF, 1 mM DTT, and complete mini EDTA-free protease inhibitor cocktail (Roche). The denatured samples were loaded on 10% or 15% SDS-PAGE gels and transferred onto nitrocellulose membranes using the Transblot^®^ TurboTM RTA Transfer Kit (Biorad, Hercules, CA, USA). Ponceau S staining (Sigma-Aldrich, St Louis, MO, USA) served as loading control. Following primary and secondary antibodies were used: mouse anti-P62 (1/5000; H00008878-M01, Abnova, Taipeh, Taiwan), rabbit anti-LC3 (1/1000; NB100-2220, Novus Biologicals, Littleton, CO, USA), peroxidase-coupled goat anti-mouse (1/10000; 115-036-068, Jackson ImmunoResearch, Ely, UK), and peroxidase-coupled goat anti-rabbit (1/10000; 112-036-045, Jackson ImmunoResearch). Signal intensity was recorded with the Amersham Imager 600 (Amersham, UK).

For immunofluorescence studies, 8 µm tibialis anterior cryosections were fixed in 4% PFA and permeabilized and blocked with PBS-Triton X-100 0.3% and PBS-Triton X-100 0.1% with 5% Bovine serum albumin, respectively. Following primary and secondary antibodies used: rabbit anti-Pax-7 (1/400; PA1-117) and rat anti-KI-67 (1/500; 14-5698-12, both ThermoFisher Scientific), Alexa488-coupled goat anti-Rabbit (1/200; 115-545-205) and Alexa594-coupled goat anti-rat (1/200; A-11007, both Jackson ImmunoResearch). Nuclei and sarcolemma were stained with DAPI (1/1000) and Alexa647-coupled wheat germ agglutinin (1/200; W32466, ThermoFisher Scientific). Images were recorded using an Axio Observer Z1 microscope (Zeiss, Jena, Germany).

### 4.8. shRNA Cloning and AAV Production

shRNA sequences were designed to target *Orai1* regions conserved in human and mouse and diverging from *Orai2* and *Orai3*. For each *Orai1* shRNA, scramble shRNAs were calculated using a specific design software (https://www.invivogen.com/sirnawizard/scrambled.php, accessed on 23 April 2022). The shRNAs (Table S2) were subcloned into pENTR1A and cloned into the pAAV plasmid under the control of the U6 promoter and flanked by serotype 2 inverted terminal repeats using the Gateway system (ThermoFisher Scientific). sh190 targets the same 19 nucleotides as the SYL116011 siRNA, developed by Sylentis to treat ocular allergies and conjunctivitis [65,66].

AAV particles were produced by triple transfection of the HEK293T cell line with pAAV, the helper plasmid, and pXR1 containing rep and cap genes of AAV serotype 9. Cell lysates were treated with 50 U/mL Benzonase (Sigma-Aldrich) for 30 min at 37 °C and clarified by centrifugation. Viral particles were purified by iodixanol gradient ultracentrifugation using Amicon Ultra-15 Centrifugal Filters (Merck, Darmstadt, Germany) and followed by dialysis. Particle quantity was determined by real-time PCR using TACGGTAAACTGCCCACTTG (forward) and AGGAAAGTCCCATAAGGTCA (reverse) primers. Titers are expressed as viral genomes per mL (vg/mL).

### 4.9. shRNA Screening and Intramuscular AAV Injection

For the cellular shRNA screening, pENTR1A plasmids were transfected into C2C12 myoblasts using Lipofectamine 3000 (Invitrogen, Waltham, MA, USA). Cells were harvested after 48 h to extract RNA and quantify *Orai1* expression. For in vivo validation, 1-month-old WT mice were anesthetized by intraperitoneal injection of ketamine 100 µg/g and xylazine 5 µg/g of body weight. TAs were injected with 1.2 × 10^10^ viral genomes/TA or 20 µL of NaCl 0.9% as control. At 2 months of age, the animals were euthanized, and *Orai1* silencing in TA samples was assessed by RT-qPCR.

To evaluate the therapeutic potential of the shRNAs, 2 months old WT and Stim1^R304W/+^ mice were anesthetized and randomly injected with 1.5 × 10^10^ viral genomes/TA or 25 µL of NaCl 0.9% as control. At 4 months of age, the mice underwent in situ muscle force measurements, and the TAs were dissected for subsequent morphological and gene expression analyses.

### 4.10. Study Randomization and Statistical Analysis

All experiments were performed and analyzed in a blinded manner and the investigators were unaware of the genotype of the mice. The normal distribution of the data was assessed using the Shapiro–Wilk test and presented as mean ± standard error of the mean (SEM). For normally distributed data, the significance of changes was examined by a two-tailed Student’s t-test with or without Welch’s correction for comparison of 2 groups or by one-way ANOVA followed by Tukey’s post hoc test for comparison of more than 2 groups. In case of not-normally distributed data, the Mann–Whitney test was used to compare 2 groups and Kruskal–Wallis followed by Dunn’s multiple comparison test to compare more than 2 groups. The statistical significance of the birth ratio was assessed by a chi-square test. Significant differences are indicated as */α/$ *p* < 0.05, **/αα/$$ *p* < 0.01, ***/ααα/$$$ *p* < 0.001, and ****/αααα/$$$$ *p* < 0.0001 with * reflecting the comparison with the WT/scramble-injected WT group, α the comparison with the *Orai1^+/−^*/shRNA-injected WT group, and $ the comparison with the *Stim1^R304W/+^Orai1^+/−^*/scramble-injected *Stim1^R304W/+^* group. Bars highlight differences between two selected groups.

## Data Availability

The authors confirm that the data supporting the findings of this study are available within the article and its supplementary material.

## Supplementary Materials

The following supporting information can be downloaded at: https://www.mdpi.com/article/10.3390/ijms23136968/s1.
