# Supplementary material for: Silencing of the Ca2+ Channel ORAI1 Improves the Multi-Systemic Phenotype of Tubular Aggregate Myopathy (TAM) and Stormorken Syndrome (STRMK) in Mice
Source: Int J Mol Sci. 2022 Jun 23;23(13):6968. doi: 10.3390/ijms23136968 (PMC9266658; doi:10.3390/ijms23136968)
Supplement: Supplementary file 1 [file ijms-23-06968-s001.zip › ijms-1737257-supplementary.pdf]

ORAI1 silencing partially improves the multi-systemic phenotype of tubular aggregate myopathy and Stormorken syndrome in mice

Roberto Silva-Rojas, Emma Lafabrie, Laura Perez-Guàrdia, David Moulaert, Jocelyn Laporte, Johann Böhm

SUPPLEMENTAL MATERIAL

List of abbreviations

4-PBA	4-Phenylbutyric acid
AAV	Adeno-associated virus
AICAR	5-Aminoimidazole-4-carboxamide ribonucleotide
ANOVA	Analysis of variance
C2C12	Immortalized mouse myoblast cell line
Ca^{2+}	Calcium
CASQ1	Calsequestrin-1
CCD	Central core disease
DAPI	4′,6-diamidino-2-phenylindole
DMD	Duchenne muscular dystrophy
DTT	Dithiothreitol
EDTA	Ethylenediaminetetraacetic acid
ER/SR	Endoplasmic/sarcoplasmic reticulum
GoF	Gain-of-function
H&E	Hematoxylin and eosin
HEK293T	Human embryonic kidney 293 cells with mutant SV40 large T antigen
Hspa5	Heat shock 70 kDa protein 5

IMD	Immunodeficiency
LC3-II	Protein encoded by *Map1lc3b*
LoF	Loss-of-function
Map1lc3a/b	Microtubule-associated proteins 1A/1B light chain 3A/B
(µCT)	Micro-computerized bone tomography
MinFeret	Minimum Feret's diameter
MOI	Moment of inertia
mTORC1	Mammalian target of rapamycin complex 1
NMJ	Neuromuscular junction
OMIM	Online Mendelian Inheritance in Man
ORAI1/2/3	named after the Horai from Greek mythology
p62	Protein encoded by *Sqstm1*
PBS	Phosphate-buffered saline
PFA	Paraformaldehyde
PMSF	Phenylmethylsulfonyl fluoride
R304W	Amino acid substitution arginine (R) → tryptophane (W) at position 304
RIPA	Radio-immunoprecipitation
RNA	Ribonucleic acid
RT-qPCR	Real-time quantitative PCR (polymerase chain reaction)
Scr	Scramble
SDS-PAGE	Sodium dodecyl sulfate–polyacrylamide gel electrophoresis
SEM	Standard error of the mean
SERCA	Sarcoplasmic/endoplasmic reticulum Ca^{2+}-ATPase
shRNA	Small hairpin RNA
SOCE	Store-operated Ca^{2+} entry

Sqstm1	Sequestosome-1
STIM1	Stromal Interaction Molecule 1
STRMK	Stormorken syndrome
TA	Tibialis anterior
TAM	Tubular aggregate myopathy
TRPC	Transient receptor potential cation channel
UPR	Unfolded protein response
WT	Wild type
XBP1	X-box binding protein 1
XLCNM	X-linked centronuclear myopathy

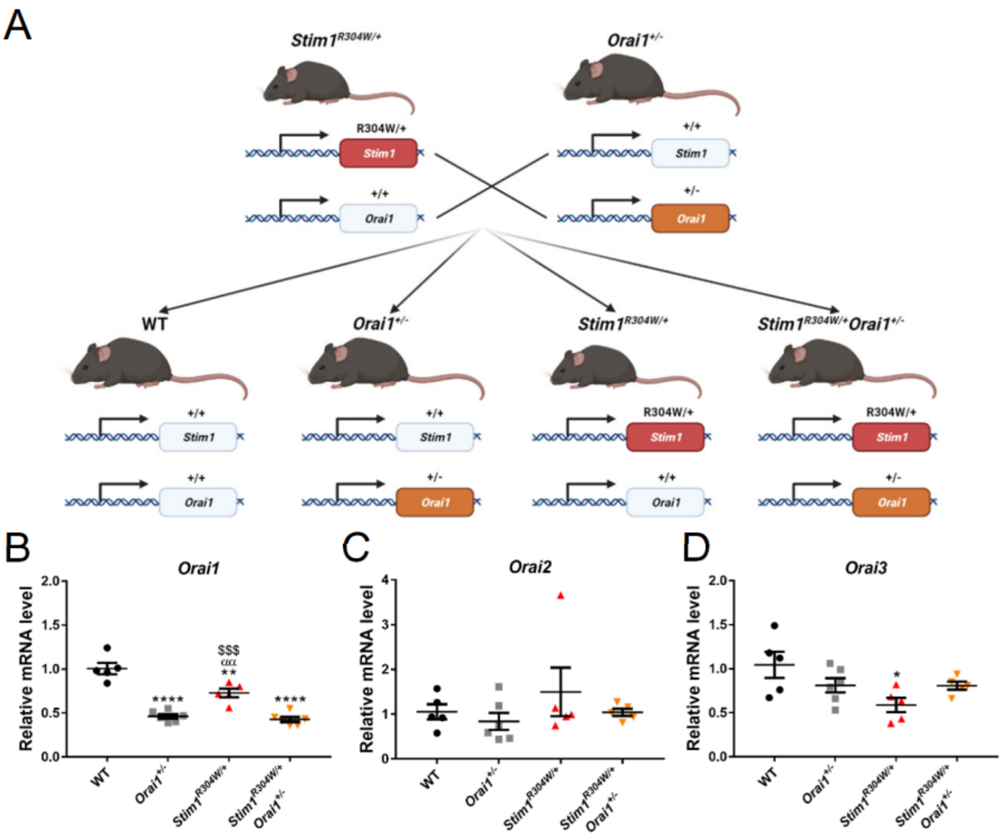

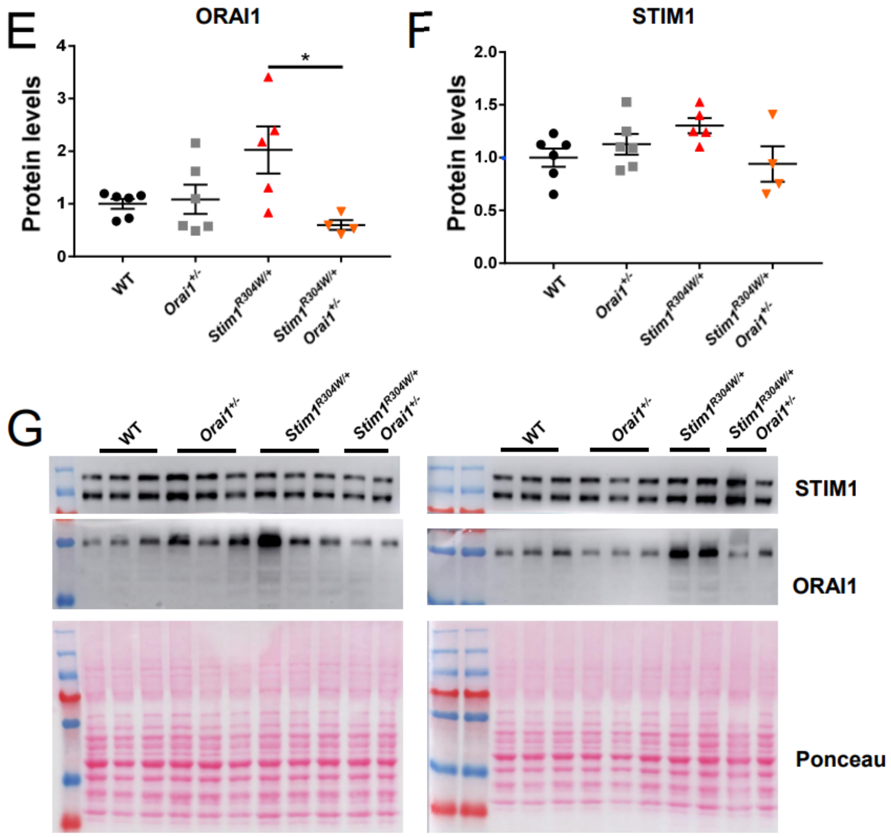

Figure S1. Crossing scheme and expression level of the SOCE key players (A) Crossing of *Stim1*$^{R304W/+}$ and *Orai1*$^{+/-}$ mice resulted in four genotypes: WT, *Orai1*$^{+/-}$, *Stim1*$^{R304W/+}$, and *Stim1*$^{R304W/+}$*Orai1*$^{+/-}$. (B-D) RT-qPCR evidenced reduced *Orai1* expression in *Orai1*$^{+/-}$ and *Stim1*$^{R304W/+}$*Orai1*$^{+/-}$ offspring compared with controls, *Orai2* expression was comparable across the genotypes, and *Orai3* expression was slightly decreased in *Stim1*$^{R304W/+}$mice (n=5-6). (E-G) Western blot on muscle extracts revealed decreased ORAI1 protein levels in *Stim1*$^{R304W/+}$*Orai1*$^{+/-}$ mice compared with WT and *Stim1*$^{R304W/+}$littermates, while STIM1 protein levels remained unchanged (n=4-6). .

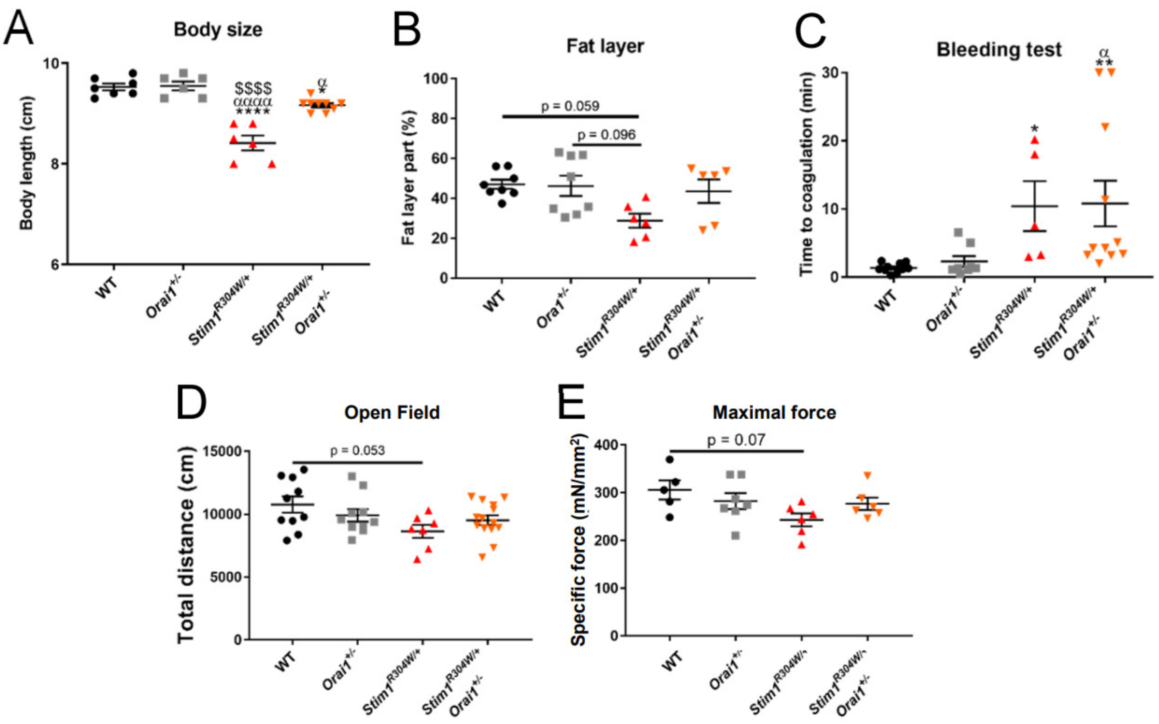

Figure S2. Body size, skin architecture, coagulation, open field test, and muscle force in *Stim1$^{R304W/+}$Orai1$^{+/-}$* mice. (A) *Stim1$^{R304W/+}$Orai1$^{+/-}$* mice were larger than the *Stim1$^{R304W/+}$* littermates at 4 months (n=6-9). (B) Statistically indistinguishable fat layer diameter in WT, *Orai1$^{+/-}$*, *Stim1$^{R304W/+}$*, and *Stim1$^{R304W/+}$Orai1$^{+/-}$* skin samples at 8 months of age, but improvement in a subset of the *Stim1$^{R304W/+}$Orai1$^{+/-}$* animals (n=5-8). (C) Increased coagulation time in *Stim1$^{R304W/+}$* and *Stim1$^{R304W/+}$Orai1$^{+/-}$* mice at 2 months compared to healthy controls (n=5-11). (D,E) Non-significant tendencies of increased covered distance in the open field arena, and maximal muscle force of *Stim1$^{R304W/+}$Orai1$^{+/-}$* mice compared with *Stim1$^{R304W/+}$* controls (n=5-14). Graphs represent mean ± SEM. Significant differences are indicated as */α/$ P<0.05, **/αα/$$ P<0.01, ***/αααα/$$$ P<0.001, and ****/αααα/$$$$ P<0.0001 with * reflecting the comparison with the WT group, α the comparison with the *Orai1$^{+/-}$* group, and $ the comparison with the *Stim1$^{R304W/+}$Orai1$^{+/-}$* group.

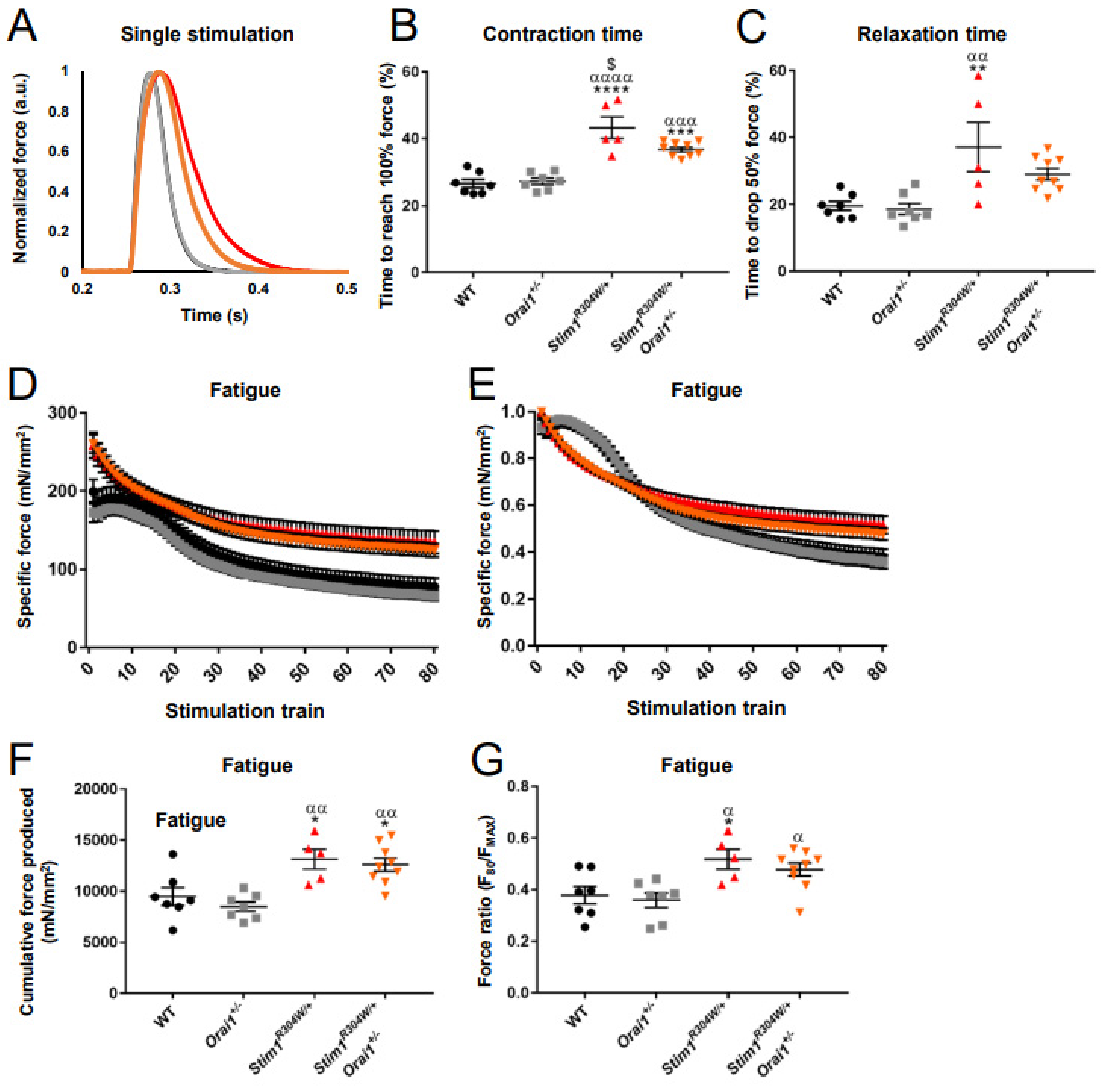

Figure S3. Improved muscle contraction properties in *Stim1*$^{R304W/+}$*Orai1*$^{+/-}$ mice. (A-C) Significantly faster muscle contraction and non-significant tendency of improved muscle relaxation following single stimulations in *Stim1*$^{R304W/+}$*Orai1*$^{+/-}$ compared with *Stim1*$^{R304W/+}$ mice at 4 months (n=5-9). (D,E) Specific and normalized force produced across 80 stimulation trains of 40 Hz illustrate different fatigue curves of *Stim1*$^{R304W/+}$ and *Stim1*$^{R304W/+}$*Orai1*$^{+/-}$ muscle compared with healthy controls at 4 months (n=5-9). (F) Quantification of fatigue as the cumulation of force

following 80 stimulations (n=5-9). (G) Quantification of fatigue as the ratio between the last and the highest force level within the stimulation train (n=5-9). Graphs represent mean ± SEM. Significant differences are indicated as */α/$ P<0.05, **/αα/$$ P<0.01, ***/ααα/$$$ P<0.001, and ****/αααα/$$$$ P<0.0001 with * reflecting the comparison with the WT group, α the comparison with the *Orai1*$^{+/-}$ group, and $ the comparison with the *Stim1*$^{R304W/+}$*Orai1*$^{+/-}$ group.

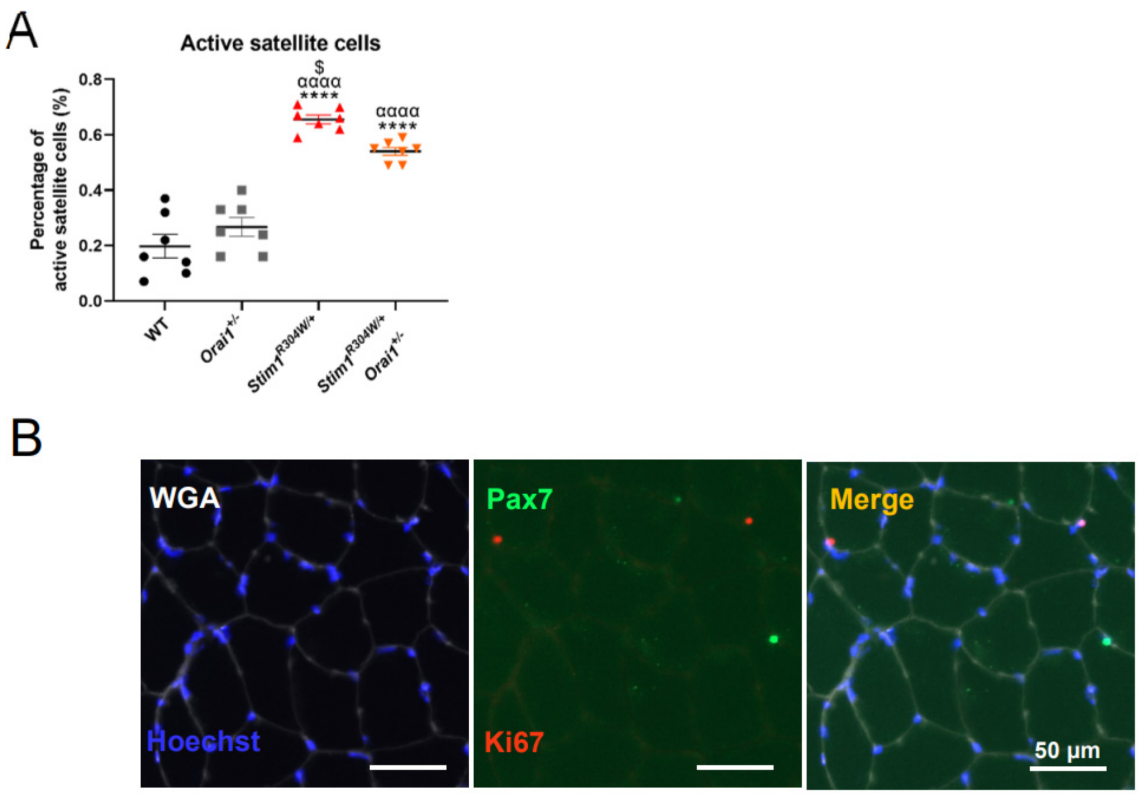

Figure S4. Satellite cell activation in *Stim1*$^{R304W/+}$*Orai1*$^{+/-}$ muscle. (A,B) The ratio of activated satellite cells was moderately but significantly reduced in *Stim1*$^{R304W/+}$*Orai1*$^{+/-}$ muscle samples compared with *Stim1*$^{R304W/+}$ littermates (n=7 per group). Representative immunofluorescence images showing Pax-7-positive satellite cells and KI-67-positive active satellite cells on

Stim1$^{R304W/+}$*Orai1*$^{+/-}$ muscle sections at 4 months. Graphs represent mean ± SEM. Significant differences are indicated as */α/$ P<0.05 and ****/αααα/$$$$ P<0.0001 with * reflecting the comparison with the WT group, α the comparison with the *Orai1*$^{+/-}$ group, and $ the comparison with the *Stim1*$^{R304W/+}$*Orai1*$^{+/-}$ group.

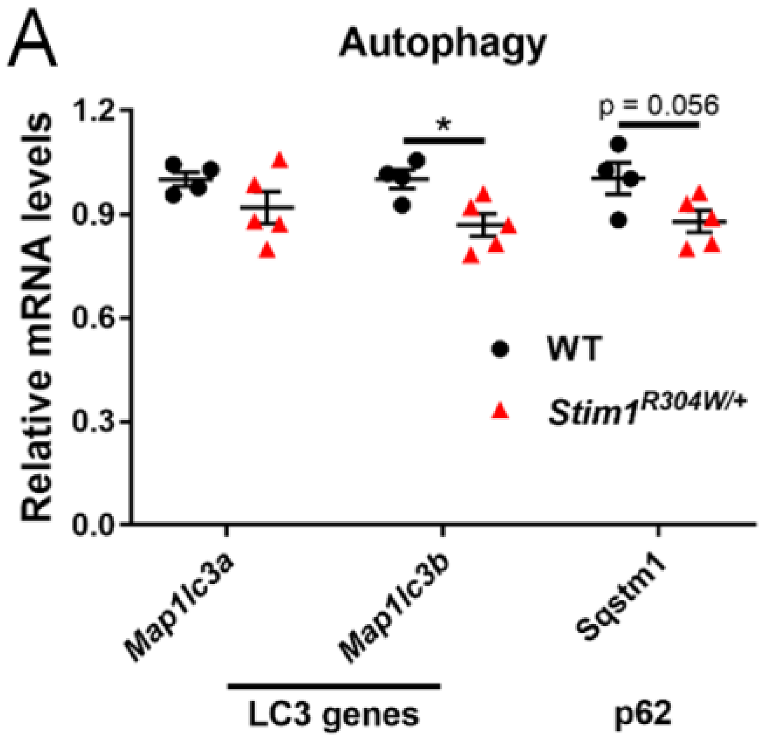

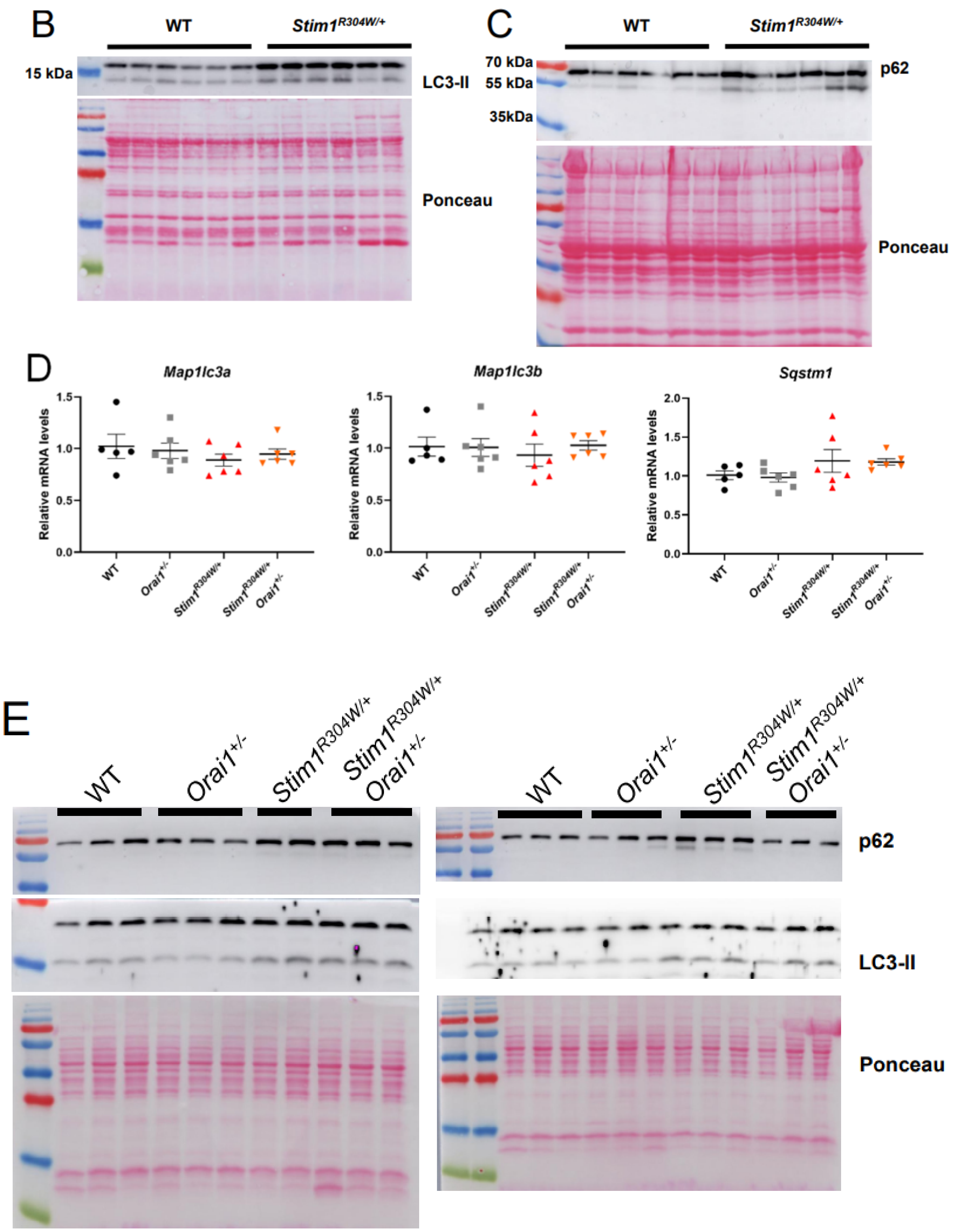
B
WT
Stim1R304W/+
15 kDa
LC3-II
Ponceau
C
WT
Stim1R304W/+
70 kDa
55 kDa
35kDa
p62
Ponceau
D
Map1lc3a
Map1lc3b
Sqstm1
Relative mRNA levels
1.5
1.0
0.5
0.0
2.0
WT
Orai1+/-
Stim1R304W/+
Stim1R304W/+ Orai1+/-
E
WT
Orai1+/-
Stim1R304W/+
Stim1R304W/+ Orai1+/-
p62
LC3-II
Ponceau

Figure S5. Partially resolved autophagy defects in *Stim1*$^{R304W/+}$*Orai1*$^{+/-}$ muscle. (A) Similar or slightly decreased *Map1lc3a*, *Map1lc3b* (both encoding LC3-II), and *Sqstm1* (p62) expression in *Stim1*$^{R304W/+}$ muscle compared with the WT at 4 months (n=4-5). (B,C) Western blots on muscle extracts evidenced intense LC3-II and p62 signals in *Stim1*$^{R304W/+}$ muscle (n=6 per group). Ponceau S staining served as loading control. (D) Similar expression of *Map1lc3a*, *Maplc3b* and *Sqstm1* across the genotypes (n-5-6). (E) Western blots revealed decreased expression of lipidated LC3 (LC3 II) and p62 in *Stim1*$^{R304W/+}$*Orai1*$^{+/-}$ muscle compared with *Stim1*$^{R304W/+}$ mice (n=5-6). Ponceau S staining served as loading control. Graph represents mean ± SEM. Significant differences are indicated as * P<0.05, with * reflecting the comparison with the WT group.

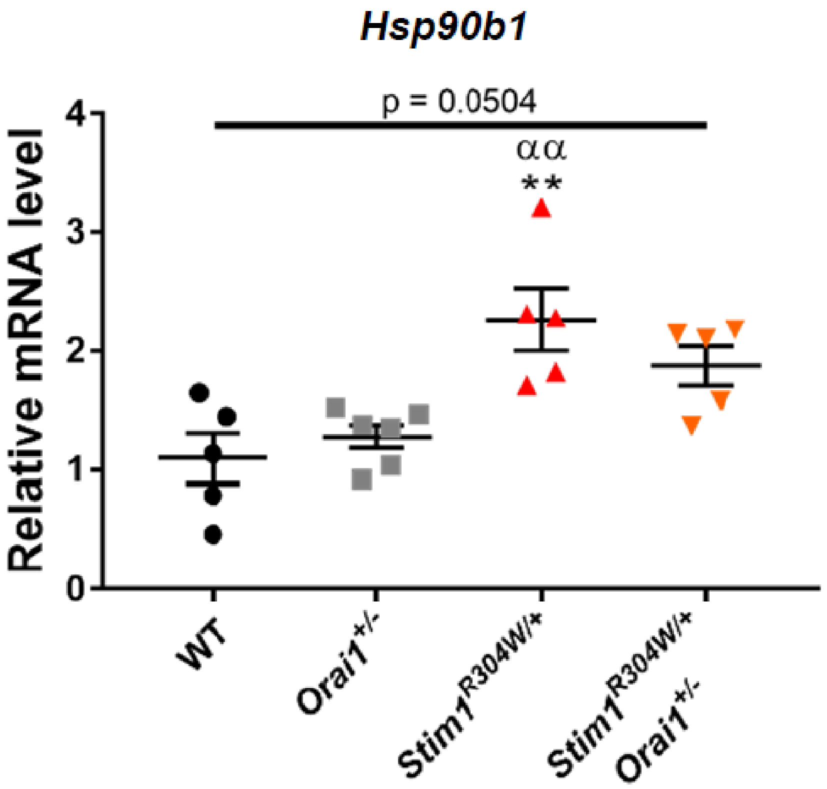

Figure S6. *Hsp90b1* expression in *Stim1*$^{R304W/+}$*Orai1*$^{+/-}$ tibialis anterior. RT-qPCR revealed an increased expression of *Hsbp90b1* in *Stim1*$^{R304W/+}$ and *Stim1*$^{R304W/+}$*Orai1*$^{+/-}$ muscle samples (n=5-6). Graphs represent mean ± SEM. Significant differences are indicated as **/αα/$$ P<0.01 with * reflecting the comparison with the WT group, and α the comparison with the *Orai1*$^{+/-}$ group.

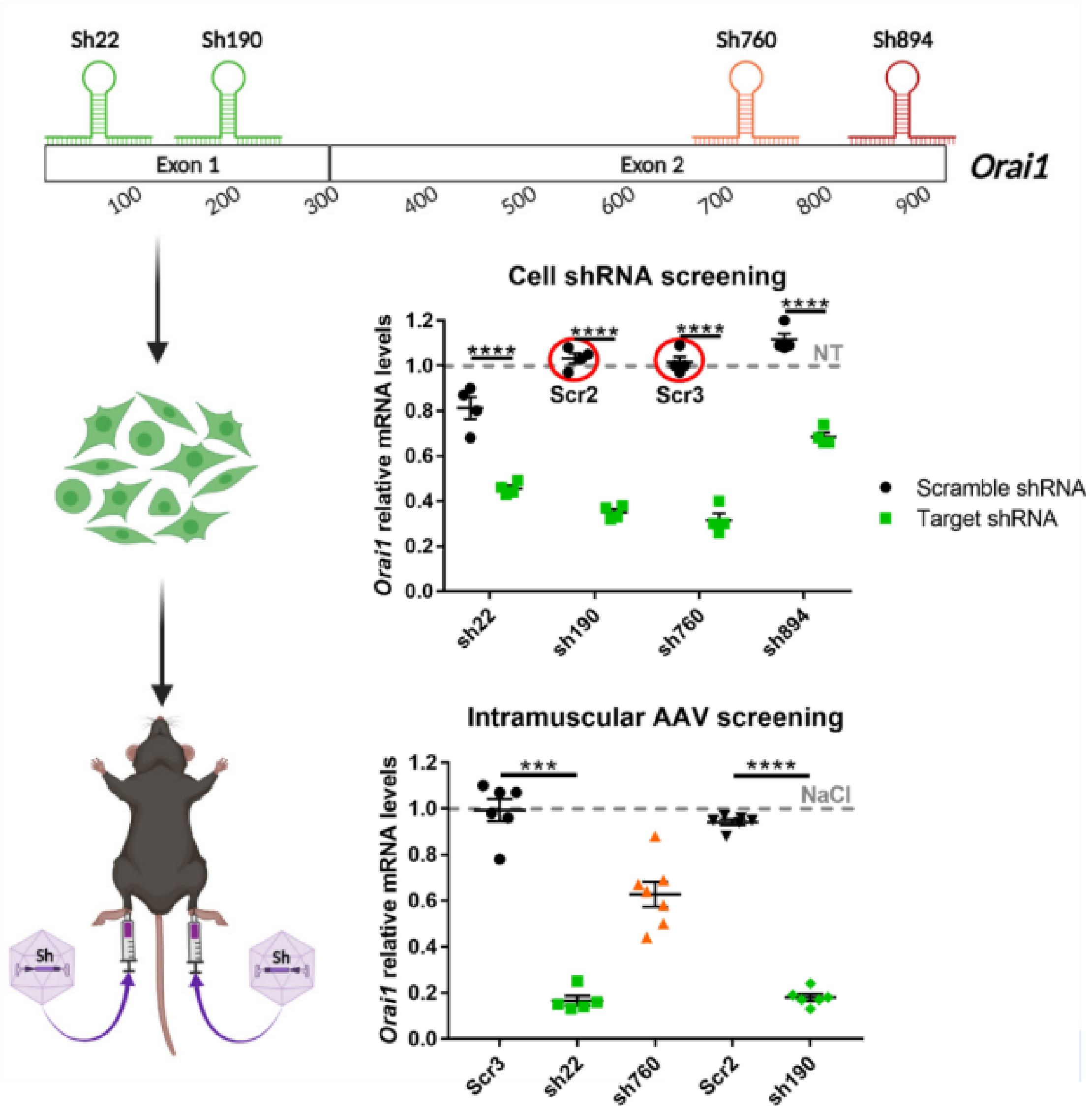

Figure S7. *In cellulo* and *in vivo* validation of shRNAs. Schematic representation of *Orai1* mRNA and positions targeted by the shRNAs. sh22, sh190 and sh760 efficiently reduced *Orai1* expression in transfected C2C12 cells, while scrambles 2 and 3 (Scr2, Scr3) had no effect (n=4 per group). The dashed line reflects the *Orai1* expression level in untreated cells. AAV9 particles containing the shRNAs were injected into the tibialis anterior of 1-month old mice. sh22 and sh190 yielded a reduction of *Orai1* expression of 80 % compared with scramble shRNAs and NaCl treatment (dashed line) 4 weeks post injection (n=5-7). Graphs represent mean ± SEM. Significant differences are indicated as *** P<0.001 and **** P<0.0001 with * reflecting the comparison with the scramble-injected group.

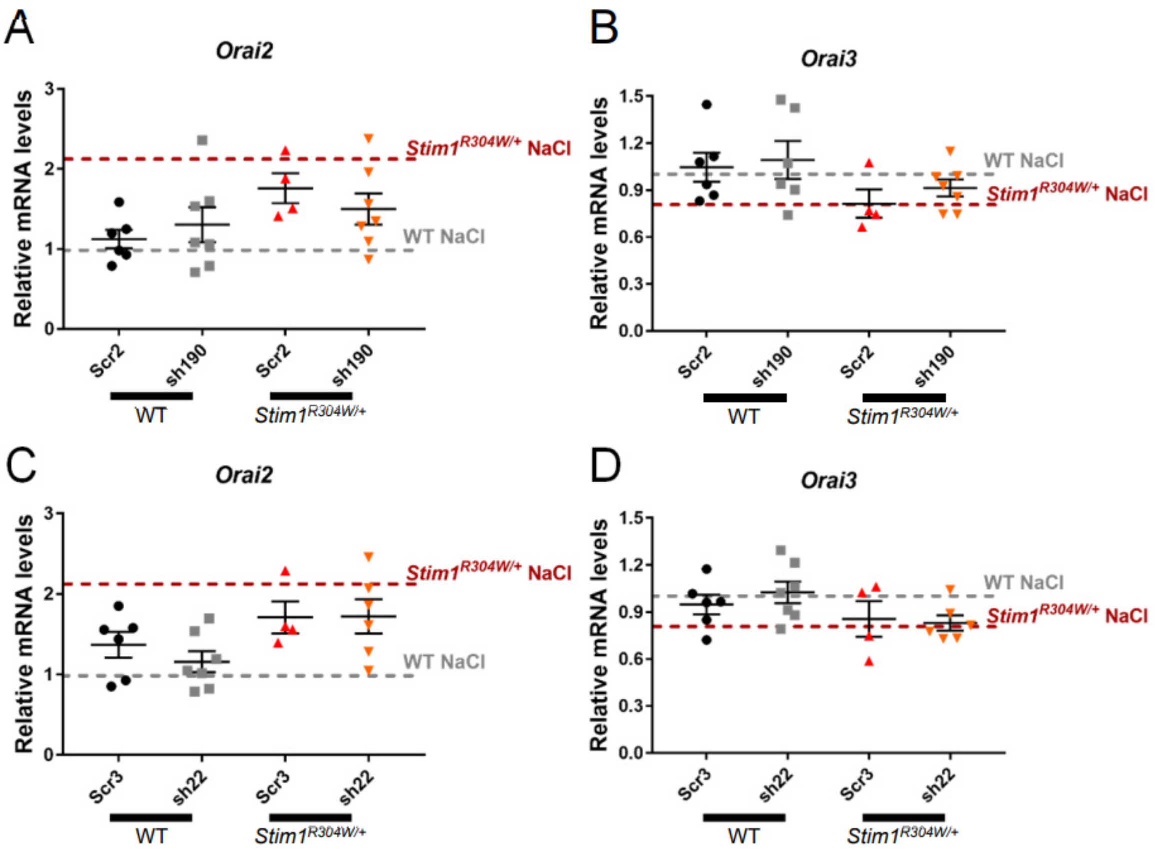

Figure S8. Normal *Orai2* and *Orai3* expression following shRNA treatment. (A-D) Comparable *Orai2* and *Orai3* expression levels in *Stim1*$^{R304W/+}$ muscle treated with *Orai1*-specific shRNAs, scramble shRNAs, or NaCl 2 months post injection (n=4-7). Graphs represent mean ± SEM.

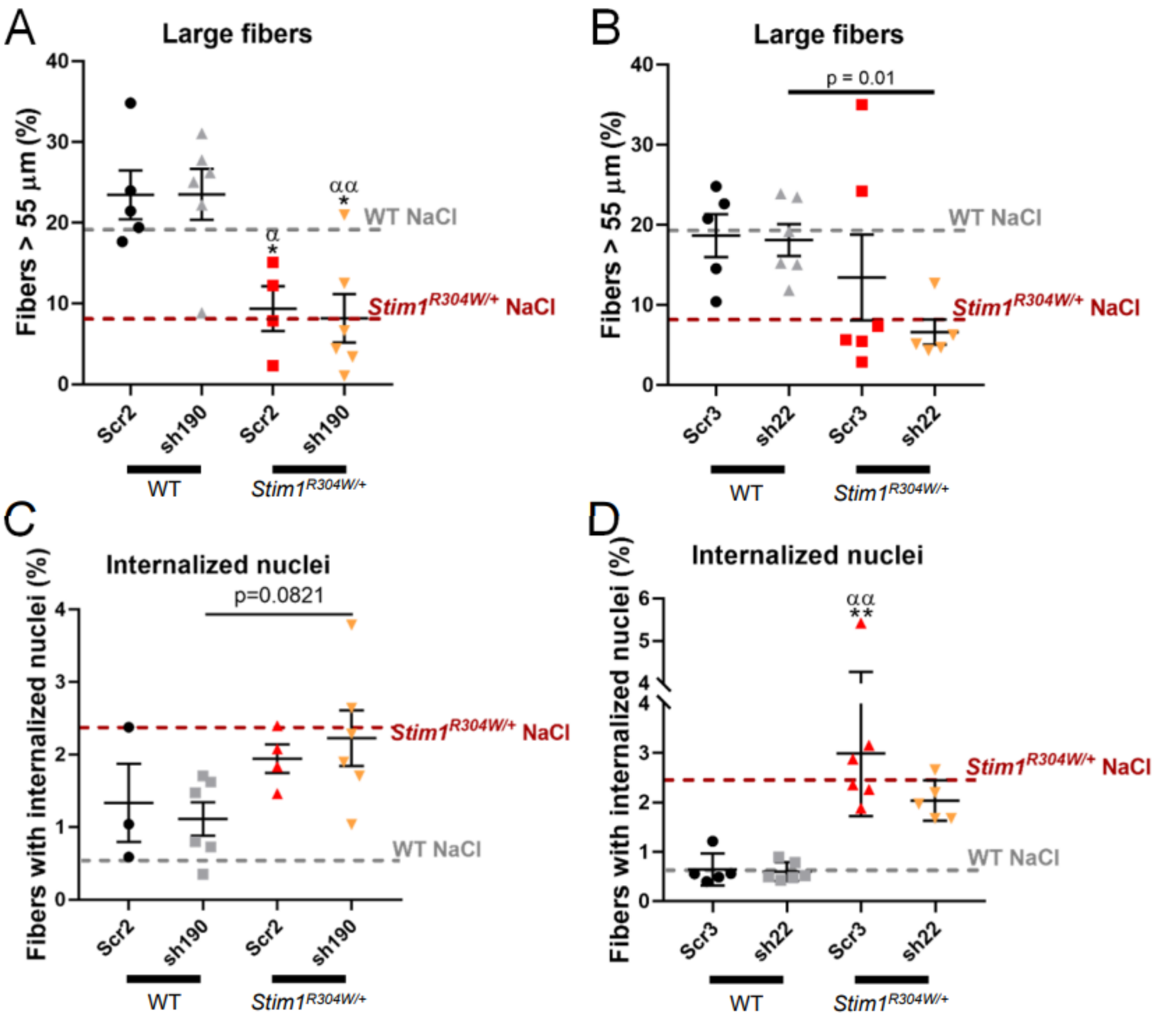

Figure S9. No effect of sh22 and sh190 on fiber size and muscle degeneration. (A-D) The low percentage of large fibers and the increased proportion of myofibers with central nuclei in

Stim1$^{R304W/+}$ muscle was not rescued by shRNA treatment 2 months post injection (n=3-7). Graphs represent mean ± SEM. Significant differences are indicated as */α P<0.05 and **/αα P<0.01 with * reflecting the comparison with the scramble-injected WT group and α the comparison with the shRNA-injected WT group.

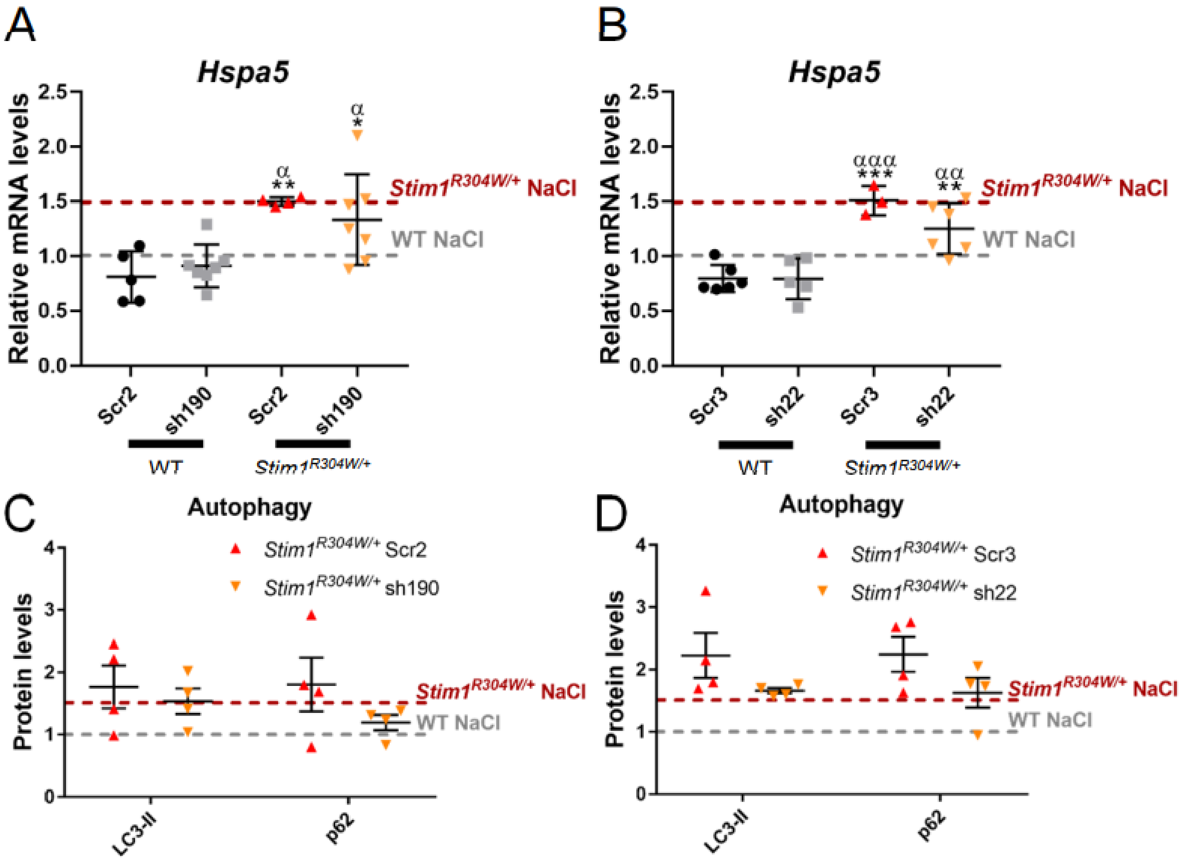

Figure S10. No effect of sh22 and sh190 on UPR and autophagosome accumulation. (A,B) Comparable expression of the UPR marker *Hspa5* in treated and untreated *Stim1*$^{R304W/+}$ muscle 2 months post injection (n=3-7). (C,D) Comparable protein levels of lipidated LC3 (LC3-II) and p62 in treated and untreated *Stim1*$^{R304W/+}$ muscle 2 months post injection (n=4 per group). Graphs represent mean ± SEM. Significant differences are indicated as */α P<0.05, **/αα P<0.01, and

***/ααα/$$$ P<0.0001 with * reflecting the comparison with the scramble-injected WT group and α the comparison with the shRNA-injected WT group.

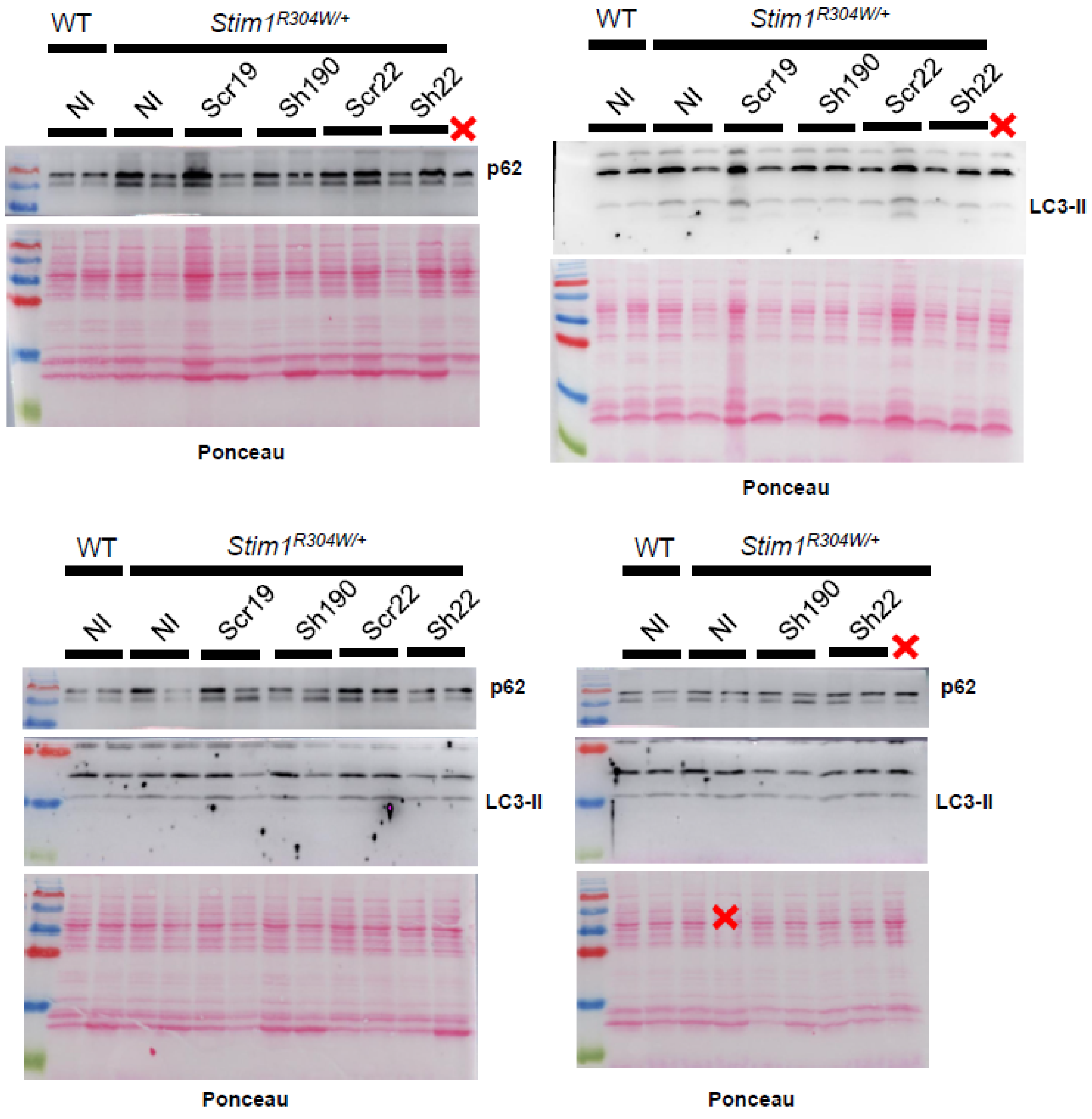

Figure S11. LC3-II and p62 protein levels. Western blots indicated comparable LC3-II and p62 signal intensities in extracts from treated and untreated *Stim1*$^{R304W/+}$ muscles 2 months post injection (n=4 per group). Ponceau S staining served as loading control.

Table S1. List of primers used for RT-qPCR.

Gene	Forward primer	Reverse primer
Rpl27	AAGCCGTCATCGTGAAGAACA	CTTGATCTTGGATCGCTTGGC
Orai1	GCCAAGCTCAAAGCTTCC	CCTGGTGGGTAGTCATGGTC
Orai2	GCAGCTACCTGGAACTCGTCACG	GAGGGTACTGGTACTTGGTCTCCA
Orai3	GGGCCAGTCAGCACTCTC	AGTGGTGCAGGCACTAAATG
Hspa5	CTATTCCTGCGTCGGTGTGT	ATTCCAAGTGCGTCCGATGA
Hspb90b1	CCACTCAAATCGAACACGGC	AGATTCCGCCTCCTTTCTGC
uXbp1	CAGACTATGTGCACCTCTGC	CAGGGTCCAACTTGTCCAGAAT
sXbp1	GCTGAGTCCGCAGCAGGT	CAGGGTCCAACTTGTCCAGAAT
Map1lc3a	CTATGAACAGGAGAAGGATGAAG	ACTCAGAAGCCGAAGGTT
Map1lc3b	CGTCCTGGACAAGACCAAGT	ATTGCTGTCCCGAATGTCTC
Sqstm1	CCTTGCCCTACAGCTGAGTC	CACACTCTCCCCCACATTCT

Table S2. Oligonucleotides used for shRNA cloning. Underlined sequences indicate the shRNA backbone hybridizing to the *Orai1* mRNA.

Oligo name	Sequence
Scr1_Fw	AGCTTTGTTTACGACGTCACGGCAGCGATCACTTCAAGAGAGTGATCGCTGCCGTGACGTCGTCTTTTTA
Scr1_Rv	GATCTAAAAAGACGACGTCACGGCAGCGATCACTCTCTTGAAGTGATCGCTGCCGTGACGTCGTAAACAA
Sh22_Fw	AGCTTTGTTT**G**CCCGAGTCACAGCAATCCGGATTCAAGAGATCCGGATTGCTGTGACTCGGG**C**CTTTTTA
Sh22_Rv	GATCTAAAAAG**G**CCCGAGTCACAGCAATCCGGATCTCTTGAATCCGGATTGCTGTGACTCGGG**C**AAACAA
Scr19_Fw	AGCTTTGTTTTAGGCGCGTCACGAAGAACTTCAAGAGAGTTCTTCGTGACGCGCCTACTTTTTA
Scr19_Fw	GATCTAAAAAGTAGGCGCGTCACGAAGAACTCTCTTGAAGTTCTTCGTGACGCGCCTAAAACAA
Sh190_Fw	AGCTTTGTTT**G**GATGAGCCTCAACGAGCATTCAAGAGATGCTCGTTGAGGCTCATC**C**CTTTTTA
Sh190_Rv	GATCTAAAAAG**G**GATGAGCCTCAACGAGCATCTCTTGAATGCTCGTTGAGGCTCATC**C**AAACAA
Scr22_Fw	AGCTTTGTTTTCTTATGCGGTATTCTCTCTTCTTCAAGAGAGAAGAGAGAATACCGCATAAGACTTTTTA
Scr22_Rv	GATCTAAAAAGTCTTATGCGGTATTCTCTCTTCTCTCTTGAAGAAGAGAGAATACCGCATAAGAAAACAA
Sh760_Fw	AGCTTTGTTTATCGTCTTTGCTGTTCACTTCTTTCAAGAGAAGAAGTGAACAGCAAAGACGATCTTTTTA
Sh760_Rv	GATCTAAAAAGATCGTCTTTGCTGTTCACTTCTTCTCTTGAAAGAAGTGAACAGCAAAGACGATAAACAA
Scr4_Fw	AGCTTTGTTTGACCACACAGTCGCGCTATACCTTCAAGAGAGGTATAGCGCGACTGTGTGGTCCTTTTTA
Scr4_Rv	GATCTAAAAAGGACCACACAGTCGCGCTATACCTCTCTTGAAGGTATAGCGCGACTGTGTGGTCAAACAA
Sh894_Fw	AGCTTTGTTTACCGGGCACCCACTATGCCTAATTCAAGAGATTAGGCATAGTGGGTGCCCGGTCTTTTTA
Sh894_Rv	GATCTAAAAAGACCGGGCACCCACTATGCCTAATCTCTTGAATTAGGCATAGTGGGTGCCCGGTAAACAA

Table S3. Trabecular bone parameters of femur. BV/TV, bone volume fraction; Tb.Th, trabecular thickness; Tb.N, trabecular number; Tb.Sp, trabecular separation. P values refer to the comparison of *Stim1*$^{R304W/+}$ and *Stim1*$^{R304W/+}$*Orai1*$^{+/-}$ by Tukey's post hoc test one-way ANOVA of all groups (n=6-7).

	BV/TV (%)	Tb.Th (µm)	Tb.N (1/mm)	Tb.Sp (µm)
WT (n=6)	7.61 ± 1.36	68.71 ± 3.01	1.08 ± 0.15	377.60 ± 40.76
Orai1$^{+/-}$ (n=6)	4.74 ± 1.21	60.93 ± 4.54	0.73 ± 0.14	390.86 ± 22.18
Stim1$^{R304W/+}$ (n=6)	0.72 ± 0.25	51.60 ± 3.46	0.13 ± 0.04	781.76 ± 24.58
Stim1$^{R304W/+}$*Orai1*$^{+/-}$ (n=7)	3.60 ± 0.79	62.07 ± 2.96	0.55 ± 0.10	520.74 ± 36.42
p value disease vs therapy	0.1883	0.4805	0.0645	<0.0001

Table S4. Cortical bone parameters of midshaft tibia. Ct.Th, cortical thickness; T.Ar, total area; B.Ar, bone area; BA/TA, bone area fraction; M.Ar, marrow area; MOI, polar moment of inertia. P values refer to the comparison of *Stim1*$^{R304W/+}$ and *Stim1*$^{R304W/+}$*Orai1*$^{+/-}$ by Tukey's post hoc test one-way ANOVA of all groups (n=6-7).

	Ct.Th (mm)	T.Ar (mm^2)	B.Ar (mm^2)	BA/TA (%)	M.Ar (mm^2)	MOI (mm^4)
WT (n=6)	0.25 ± 0.01	0.91 ± 0.03	0.74 ± 0.03	81.09 ± 0.47	0.17 ± 0.00	0.15 ± 0.01
Orai1$^{+/-}$ (n=6)	0.23 ± 0.01	0.84 ± 0.04	0.67 ± 0.04	79.48 ± 0.56	0.17 ± 0.00	0.13 ± 0.01
Stim1$^{R304W/+}$ (n=6)	0.22 ± 0.01	0.73 ± 0.03	0.57 ± 0.03	78.64 ± 0.83	0.15 ± 0.00	0.09 ± 0.00
Stim1$^{R304W/+}$*Orai1*$^{+/-}$ (n=7)	0.25 ± 0.01	0.84 ± 0.02	0.68 ± 0.02	80.98 ± 0.32	0.16 ± 0.00	0.12 ± 0.00
p value disease vs therapy	0.0348	0.0640	0.0575	0.0304	>0.9999	0.1488
